# Research on Bi-Objective Optimization of Injection Molding Process and Mechanical Anisotropy of Glass Fiber-Reinforced Polypropylene Fan Face Shell Based on RSM and NSGA-II

**DOI:** 10.3390/polym18111373

**Published:** 2026-05-31

**Authors:** Ming Yang, Sailong Yan, Jubao Liu, Feng Li, Jianfeng Yao, Yasheng Li

**Affiliations:** 1 Mechanical Science and Engineering College, Northeast Petroleum University, Daqing 163318, China; yangm@nepu.edu.cn (M.Y.);; 2 Hongfeng Intelligent Manufacturing (Shenzhen) Co., Ltd., Shenzhen 518117, China

**Keywords:** glass fiber-reinforced polypropylene, injection molding, response surface methodology, NSGA-II, mechanical anisotropy, molding-structure co-simulation

## Abstract

Large glass fiber-reinforced polypropylene (GF-PP) shells are widely used in HVAC and automotive industries, but their injection molding suffers from severe warpage deformation, residual stress concentration, and inaccurate mechanical performance prediction due to neglected molding history. This study proposes an integrated optimization framework for a 30% GF-PP fan face shell. The optimal two-gate molding configuration was determined via Moldflow simulation. A Central Composite Design (CCD) combined with NSGA-II was used to optimize process parameters for minimizing warpage and residual stress. A Moldflow-Ansys co-simulation process was established to characterize fiber orientation-induced mechanical anisotropy, and full-scale mold trials were conducted for validation. The optimized process reduced maximum warpage by 58.03% (from 5.299 mm to 2.224 mm) and residual stress by 13.67% (from 54.93 MPa to 47.42 MPa). The average tensile modulus along the flow direction was 1.85 times that perpendicular to the flow direction. Mold trial results showed a warpage prediction error of only 7.583%. The proposed framework effectively addresses the critical quality issues in large GF-PP injection molding, providing a systematic engineering solution for molding quality control and accurate performance characterization.

## 1. Introduction

Glass fiber-reinforced polypropylene (GF-PP) composites have emerged as the primary materials for replacing metal structural components in the fields of heating, ventilation and air conditioning (HVAC), automotive industry, and home appliance manufacturing due to their low density, high specific strength, excellent chemical corrosion resistance, moldability and recyclability, with their application scale in industrial shell products continuously expanding [1]. With the development of industrial products towards lightweight and integration, the structural complexity of large-scale thin-walled GF-PP injection-molded shells is continuously increasing, which imposes more stringent requirements on the molding accuracy, dimensional stability and long-term service mechanical properties of plastic parts [2].

Injection molding is the core processing technology for GF-PP structural parts. Its process involves strong multi-physical field coupling effects such as melt flow, heat and mass transfer, fiber orientation evolution, and curing shrinkage. The final molding quality of plastic parts is synergistically affected by multiple process parameters, including melt temperature, mold temperature, holding pressure, holding time and injection rate, and there are significant nonlinear and strong coupling relationships between process parameters and molding quality [3]. For large-scale flat thin-walled GF-PP shells with complex features such as stiffeners, screw bosses and ribs, the melt has a long filling path and poor flow balance, leading to molding defects including excessive warpage deformation, weld lines and residual stress concentration. Among them, warpage deformation directly determines the assembly accuracy of plastic parts, while residual stress significantly affects the structural stiffness and service life of plastic parts. Both are core indicators for evaluating the molding quality of large-scale GF-PP shells and also key problems to be solved urgently in the injection molding process of such products.

A large number of relevant studies have been carried out by scholars on the optimization of injection molding process parameters [4,5,6]. As an efficient statistical modeling method, Response Surface Methodology (RSM) can construct a nonlinear mapping relationship between process parameters and molding quality indicators with a small number of experiments through scientific experimental design, which effectively solves the problems of high cost and low efficiency of the traditional trial-and-error method and has been widely used in the field of injection molding [7,8]. Nguyen et al. [9] numerically analyzed the warpage phenomenon inside insert injection-molded frame components and determined the optimal processing conditions based on the Taguchi method combined with RSM. The results showed that this combined technology can overcome the limitations of the Taguchi method caused by its discrete optimization characteristics, and can effectively provide more accurate optimal solutions without complex algorithms and software. Chuang et al. [10] combined RSM with statistical techniques to establish mathematical prediction models for warpage and tensile stress properties, so as to investigate the influence of injection molding parameters on warpage and tensile stress properties in the production of thin-shell plastic parts, determine the optimal conditions, and identify the most significant factors through analysis of variance (ANOVA). Berti and Monti [11] proposed a novel method integrating numerical simulation, RSM and stochastic simulation, and realized the robust optimization of the injection molding process relying on the Virtual Prototype Environment (VPE). The core of this method is to incorporate the influence of process parameter fluctuations into the numerical settings. Taking the injection molding of an engine hood as the application scenario, this study verified that VPE can achieve robust process settings considering process fluctuations, and the measurement results of small-batch trial production confirmed the accuracy of numerical prediction. Tzeng et al. [12] analyzed the influence of the injection molding process on the mechanical properties of short glass fiber and polytetrafluoroethylene reinforced polycarbonate composite blends, and proposed a hybrid method combining Back-Propagation Neural Network (BPNN), Genetic Algorithm (GA) and RSM to optimize injection molding process parameters. In this study, samples were prepared based on the Taguchi orthogonal array, and BPNN was trained with experimental data and optimized by combining GA and RSM, which verified that both methods can effectively optimize injection molding process parameters. However, RSM has limitations in dealing with multi-objective and strongly conflicting optimization problems, and it is difficult to realize the collaborative optimization of multiple mutually restrictive quality indicators. The Non-Dominated Sorting Genetic Algorithm II (NSGA-II) introduces non-dominated sorting, crowding distance calculation and elitist preservation strategies, and exhibits excellent global optimization ability in multi-objective optimization problems. Its combination with RSM has become the mainstream technical route for multi-objective optimization of injection molding processes. Li et al. [13] took the warpage, volume shrinkage and residual stress of short fiber reinforced composite injection-molded parts as quality objectives, and fiber content, aspect ratio and injection molding process parameters as design variables, and proposed a hybrid method combining the Taguchi method, RSM and NSGA-II to optimize the injection molding process. In this study, the parameter influences were clarified through orthogonal experiments, Moldflow simulation, and ANOVA. The nonlinear relationship between parameters and quality objectives was constructed using the response surface model, and the optimal parameters were obtained by combining NSGA-II, which verified the effectiveness of this hybrid method in multi-objective quality optimization. Zhao and Li [14] constructed a response surface model between controllable factors, noise factors and quality objectives, and carried out multi-objective robust optimization design by combining NSGA-II, 6 Sigma optimization design, and uncertainty analysis methods. Taking the automotive navigation fixing base as an application case, numerical simulation and actual injection molding experiments verified that this method effectively reduced the mean square error and significantly improved the robustness and safety of products compared to deterministic optimization. Tian et al. [15] proposed a two-stage systematic optimization method for the parameter optimization of the Powder Injection Molding (PIM) process. In the first stage, the Taguchi method combined with CAE simulation was used to preliminarily screen key parameters such as melt temperature and injection speed and determine their significance order. In the second stage, based on the narrowed parameter range, RSM and NSGA-II were combined to optimize multiple objectives, including dimensional accuracy, warpage, weight and energy consumption. The verification results showed that this method can not only improve process stability and dimensional accuracy but also effectively reduce product weight and production energy consumption. Wu et al. [16] proposed a method combining multi-objective optimization and Moldflow simulation to control the nonlinear shrinkage of small-module plastic gears in micro-injection molding. Taking various shrinkage indicators as optimization objectives, this study analyzed the relationship between process parameters and objectives through the quadratic response surface model (RSM-Quadratic) and performed multi-objective optimization with NSGA-II, which significantly reduced the gear shrinkage and tooth profile deviation and verified the application potential of this method in the manufacture of high-precision small-module plastic gears. Although the hybrid RSM-NSGA-II framework has become the mainstream technical route for multi-objective optimization of injection molding processes, existing studies represented by Li et al. [13] still have three critical limitations: their research objects are mostly concentrated on small and precision plastic parts with simple geometries; no strongly conflicting collaborative optimization of warpage deformation and residual stress has been carried out for large-scale flat GF-PP shells, which are characterized by extremely long melt flow paths and complex stiffener/rib features; and the coupling contradiction between the two core quality indicators is far more pronounced than that in small plastic parts, making the optimization solutions derived from small components unable to be directly migrated. Most existing studies stop at the process parameter optimization stage, and have not systematically established the correlation between the optimized molding process and microstructural characteristics such as fiber orientation distribution as well as the induced macroscopic mechanical anisotropy, thus failing to comprehensively evaluate the impact of process optimization on the actual service performance of plastic parts, which is particularly critical for load-bearing structural shells; the majority of studies lack a complete engineering verification chain, and Li et al. [13] and others only verified their optimization results through numerical simulation, while few have conducted full-scale injection molding trials and precision dimensional measurements for large industrial components, leaving significant uncertainty regarding the practical effectiveness of the proposed optimization methods in real manufacturing environments.

The mechanical properties of GF-PP injection-molded parts are directly determined by the fiber orientation distribution formed during the injection molding process. The high orientation of fibers along the flow direction leads to significant mechanical anisotropy of the material, which is the core factor affecting the actual service performance of plastic parts. Traditional structural mechanical simulation usually regards GF-PP injection-molded parts as isotropic homogeneous materials, completely ignoring the influence of molding history, such as fiber orientation, residual stress and weld lines, on the mechanical properties of materials, resulting in a great deviation between the predicted mechanical properties and actual engineering applications, which cannot provide reliable theoretical support for the structural design and process optimization of plastic parts. In recent years, the injection molding/structural–molding-structural mechanical co-simulation technology has gradually become a research hotspot in the performance characterization of composite injection-molded parts. By mapping the fiber orientation tensor, residual stress distribution and other information obtained from injection molding simulation to the structural mechanical simulation model, the anisotropic mechanical behavior of glass fiber-reinforced composites can be accurately characterized, and the prediction accuracy of mechanical properties can be greatly improved. Isaincu et al. [17] evaluated the influence of fiber orientation on the mechanical properties of tensile specimens under different mapping processes and mesh sizes. The fiber orientation tensor was determined by Moldflow software 2016, and the composite model was constructed by the Digimat mean-field homogenization tool. The mechanical property evaluation was carried out in Ansys finite element software 17.0, and the anisotropic properties of the material were considered by combining it with Digimat. Chai et al. [18] established a bending fatigue test simulation method considering the anisotropic properties of long glass fiber-reinforced thermoplastics (LGFTs). Firstly, an anisotropic material model of LGFT was established based on the tensile test results of LGFT samples. According to the fiber orientation and distribution of the rim obtained from the injection molding simulation, the anisotropic material properties of the LGFT rim were obtained. Then, a simulation model of the bending fatigue test platform was established and used to simulate the strength of the LGFT rim. The results showed that the model considering material anisotropy had a higher consistency between the predicted results and the experimental values and significantly improved the prediction accuracy compared to the traditional isotropic material model. Chen et al. [19] established a complete process-structure collaborative optimization method for the automotive glass fiber-reinforced polymer (GFRP) front-end module. The optimal combination of injection molding process parameters was determined through orthogonal experiments, and the warpage of the injection-molded part was finally reduced by 41.5%. Meanwhile, through the injection molding history mapping technology, the molding information, such as fiber orientation, residual stress and strain, was completely mapped to the structural mechanical simulation model. When the measured displacement of the component exceeded 0.65 mm, the average relative error between the simulation and experimental results was reduced to 11.78%, and the error dispersion was significantly reduced, which confirmed the reliability of this method in engineering applications. Fujita et al. [20] proposed a full failure process prediction method based on fracture mechanics for injection-molded short fiber-reinforced thermoplastic (IM-SFRP) sub-components. Firstly, the fracture toughness of PA66-GF50 material under different fiber orientations and different temperature and humidity environments was systematically characterized through compact tension (CT) tests, and the quantitative influence laws of fiber orientation components, ambient temperature and moisture content on the elastic modulus, tensile strength and critical strain energy release rate of the material were clarified. Then the fiber orientation tensor field predicted by Moldflow injection molding simulation was mapped to the structural finite element model. Combined with the Cohesive Zone Model (CZM), the fracture toughness calibrated by experiments and considering the difference in the skin-core structure of injection-molded parts was taken as the input parameter, and a mechanical simulation model coupling injection molding history and material progressive damage was established. The results showed that the prediction error of the ultimate failure load of typical sub-components of automotive engine mounts by this method can be controlled within 2.6%, and the prediction accuracy was significantly improved compared to the traditional Tsai-Hill criterion, which only considers failure initiation (the predicted failure load is 16~24% lower). Kohar et al. [21] established a mechanical property prediction method for multiphase composites based on the Representative Volume Element (RVE) for polypropylene-based sandwich composites used in automotive luggage compartments. Firstly, mesoscopic models of composites with different component ratios, such as PP/PET/glass fiber and PP/PET/cotton fiber, were constructed by Digimat FE software 2023.1, and an RVE finite element model containing 512,000 elements was generated. Multi-step loads were applied through the automatic performance evaluation function, and key mechanical parameters, such as elastic modulus, Poisson’s ratio, and shear modulus of the composites in different directions, were accurately calculated. Then the material model was imported into MSC.Marc/Mentat software 2023.1, the loading simulation of the luggage compartment side panel was carried out according to the PR375 standard to simulate the structural deformation behavior under 100N load, and the accuracy of the simulation model was verified by solid tensile tests (including cyclic loading tests) and standard load tests. The results showed that the deformation difference between simulation and experiment was only 3.4~4.5% for the measuring points in the main stress-bearing area, which confirmed the effectiveness of the RVE-based mesoscopic homogenization method in predicting the structural properties of multiphase composites. Perin et al. [22] proposed a single/multi-gate design optimization algorithm based on visual programming and machine learning, which provided a new way for the active regulation of fiber orientation of glass fiber-reinforced thermoplastic composite injection-molded parts. In this method, Finite Volume Method (FVM) simulation was first carried out by Moldflow software to obtain the fiber orientation tensor distribution corresponding to different gate positions; the fiber orientation information was mapped to the structural mechanical model through FVM-FEM co-simulation, the stress concentration area of the component was determined by Abaqus finite element simulation, the influence of different gate configurations on the reinforcement effect of key areas was quantified, and the feasibility and reliability of regulating fiber orientation to improve the mechanical properties of components by gate position optimization were verified through experiments. Kufel et al. [23] established an injection-structure co-simulation and performance prediction system considering multi-factor influences for basalt/glass fiber hybrid-reinforced polypropylene (BF/GF-PP) composites. Firstly, hybrid composite samples with different fiber contents were prepared by injection molding: the fiber orientation distribution was obtained by simulating the injection process with Moldex3D software 2023, and the mesoscopic model of multiphase composites was constructed based on the Mori–Tanaka mean-field homogenization theory by using the reverse engineering function of the Digimat-MF module, which clarified the synergistic reinforcement mechanism of basalt fibers and glass fibers. Then, the fiber orientation information and homogenized material parameters were imported into Ansys software to systematically explore the mechanical response of materials under different temperatures and cyclic loads. The results showed that this co-simulation method had extremely high prediction accuracy and could effectively characterize the regulation law of temperature on anisotropy. Although studies [17,18,19,20,21,22,23] have established the theoretical basis for injection molding–structural co-simulation, they have three critical limitations for large-scale flat thin-walled GF-PP industrial shells: first, most focus on standard specimens [17,21,23] and small components [18,20], lacking mature applications for shells over 500 mm with long melt flow paths; second, their research systems are fragmented, either performing single-objective optimization [19] or fixed-history mechanical simulation [17,18,20,21], ignoring the warpage–residual stress conflict and global process optimization [22]; third, they lack fine meso-mechanical homogenization for anisotropy characterization [18,19] and full-chain engineering verification covering both molding quality and service performance [17,19,23]. Therefore, there remains a lack of systematic research integrating RSM-NSGA-II bi-objective optimization, meso–macro coupled co-simulation, and comprehensive mold trial verification for such large-scale GF-PP shells.

Based on the above research background and existing problems, this paper takes a large-scale GF-PP fan face shell as the research object and carries out systematic research according to the whole-chain technical route of “bi-objective optimization of injection molding process—mechanical property co-simulation associated with molding history—mold trial production verification”. Firstly, the optimal design of the gating system and cooling system was completed through Moldflow numerical simulation, and the optimal two-gate molding configuration was determined. Subsequently, the Central Composite Design (CCD) was adopted to construct a second-order response surface model between key process parameters and warpage deformation as well as residual stress, and the Pareto optimal solution set for bi-objective optimization was solved by combining the NSGA-II algorithm to screen out the optimal process scheme that balances molding accuracy and residual stress control. On this basis, a Moldflow-Ansys co-simulation process was established based on the Helius PFA software 2023 to realize the accurate mapping of molding history information, and the fiber orientation distribution characteristics of plastic parts under the optimal process and the mechanical anisotropy induced by it were systematically analyzed. Finally, the engineering verification of the optimization scheme was completed by using a coordinate measuring machine through injection molding trial experiments. The research results of this paper can provide a systematic theoretical basis and engineering solution for the molding quality control and accurate service performance prediction of large-scale GF-PP injection-molded shells.

## 2. Materials and Methods

### 2.1. Experimental Objects and Material Properties

#### 2.1.1. Structural Parameters of the Fan Face Shell

**Figure 1:**
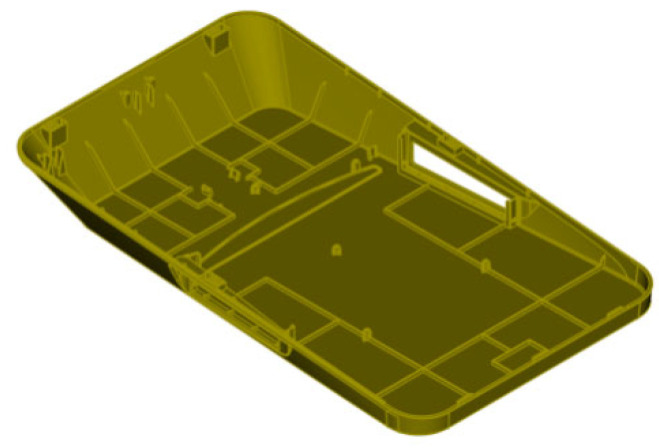
Three-dimensional model of the fan face shell.

In this study, a fan face shell is taken as the research object, with an overall dimension of 588 mm × 352 mm × 75 mm. Its three-dimensional model is shown in Figure 1, and the average wall thickness is 3 mm, which classifies it as a large-scale flat shell. The plastic part is designed with dense features such as stiffeners, rib plates, and threaded mounting bosses, which are prone to molding defects, including warpage deformation, weld lines, insufficient filling, sink marks, and excessive residual stress. Therefore, numerical simulation is required to complete the process design and optimization.

#### 2.1.2. Material Selection

**Figure 2:**
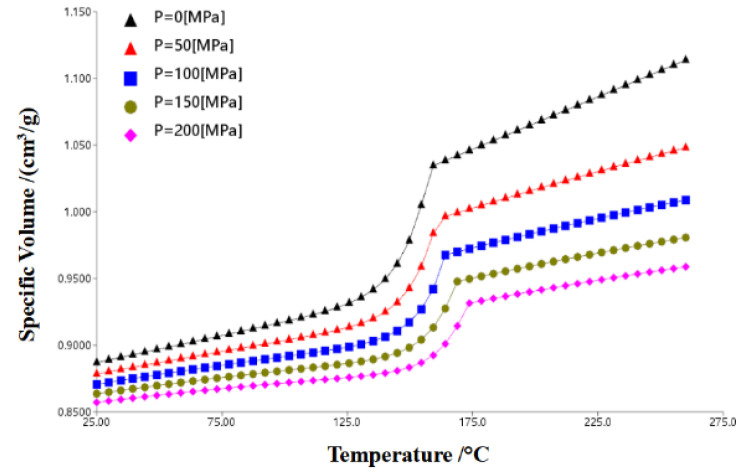
PVT curve.

**Figure 3:**
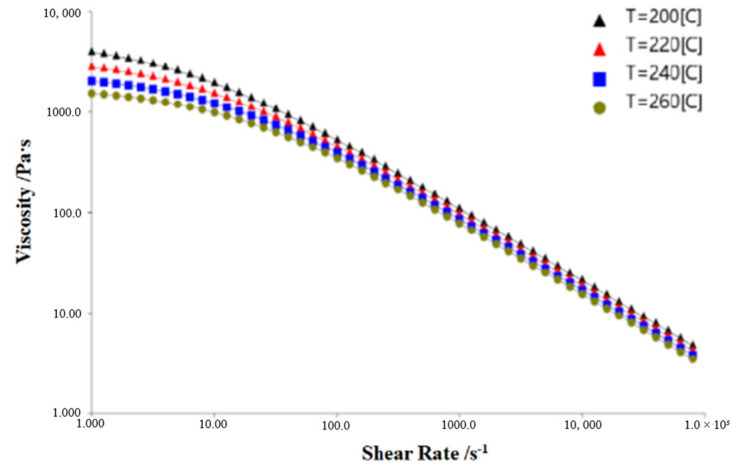
Viscosity curve.

Considering the structural characteristics, molding requirements, and service performance of the large-scale thin-walled fan face shell, a GF-PP composite was selected as the manufacturing material. The material is commercial grade Extron 3019 HS produced by Polypacific Co., Ltd. (Victoria, Australia), featuring a glass fiber mass fraction of 30% and a fiber aspect ratio of 25. The viscosity curves and pressure–volume–temperature (PVT) curves of the composite are presented in Figure 2 and Figure 3, respectively. The fundamental properties of the material and the manufacturer-recommended processing parameters are summarized in Table 1.

### 2.2. Establishment of Numerical Model for Injection Molding

#### 2.2.1. Mesh Generation

**Figure 4:**
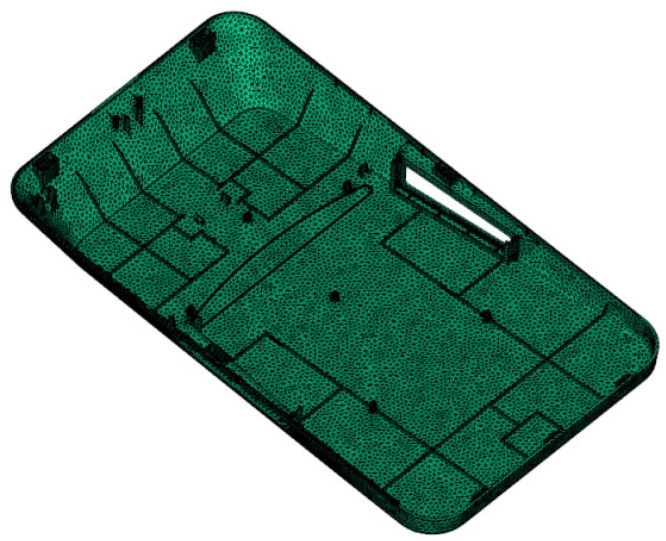
Mesh model.

Firstly, the three-dimensional model of the fan face shell was imported into CAD Doctor software 2002 to repair geometric defects such as surface flaws, holes and free edges. After the repair was completed, the model was imported into Moldflow 2023 software for mesh generation, and the mesh type was set as the dual-domain mesh. Aiming at the problems of large mesh quantity and difficult quality control of large-scale models, the built-in Advanced Import function of the software was adopted to optimize the mesh quantity on the premise of ensuring mesh matching accuracy and reducing the computational cost. After automatic mesh generation, mesh defects, including high aspect ratio, free edges, overlapping elements, and incorrect orientation, were corrected by combining the Mesh Repair Wizard with manual repair. The statistical results of the repaired mesh are listed in Table 2. The mesh matching rate reaches 93.4%, and the maximum aspect ratio is 19.03, which meets the mesh quality requirements for warpage analysis in Moldflow 2023 (matching rate ≥ 90%, maximum aspect ratio ≤ 20). Therefore, the mesh model, as shown in Figure 4, can be used for subsequent filling, warpage, and residual stress analysis.

#### 2.2.2. Design of Gating System

##### Determination of Gate Number

**Figure 5:**
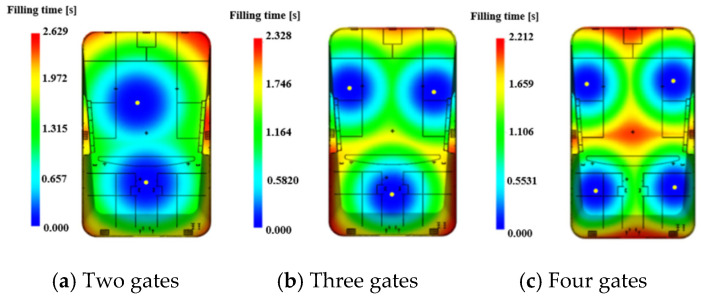
Contour plots of fill time.

**Figure 6:**
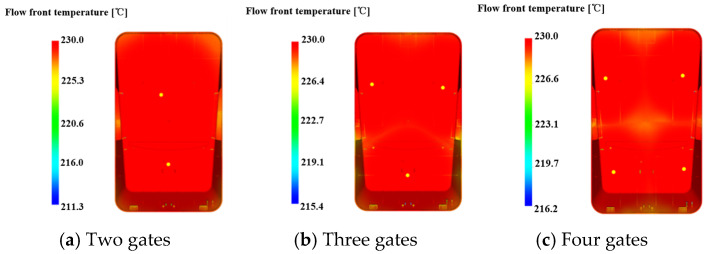
Contour plots of flow front temperature.

**Figure 7:**
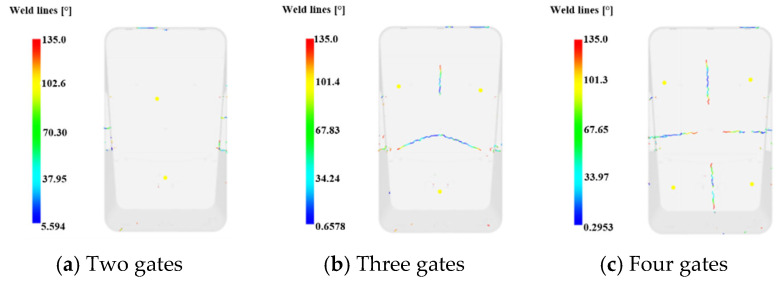
Contour plots of weld lines.

In this study, three gating system configurations with two gates, three gates, and four gates were designed. All configurations adopted the default process parameters recommended for the material, and the gate positions were automatically optimized and generated by Moldflow 2023 software based on melt flow resistance. The simulation results of fill time, flow front temperature, and weld lines for the three configurations are shown in Figure 5, Figure 6 and Figure 7.

The results show that the fill times of the two-gate, three-gate and four-gate configurations are 2.629 s, 2.328 s and 2.212 s, respectively, with a small difference in overall fill time. The temperature drops of the flow front are 4.7 °C, 3.6 °C and 3.4 °C, respectively, all within the reasonable control range of 2 °C to 5 °C. The quantitative analysis results of weld lines indicate that the number of high-risk weld lines (weld angle < 60°) on the appearance surface of the two-gate configuration is 0. In contrast, the product surfaces of the three-gate and four-gate configurations have long weld line defects. Only the two-gate configuration has fewer defects on the outer surface, and the plastic part has better appearance quality and molding uniformity. Therefore, the two-gate configuration is finally determined as the optimal gating system.

##### Dimension Calculation of the Gating System

Based on the flow theory of non-Newtonian fluids, the dimension calculation of the hot runner gating system was completed. The empirical formula for calculating the sprue diameter is shown in Equation (1):

(1) $$D_{1}=0.08\times {\left(\frac{3n_{1}+1}{n_{1}}f_{1}\right)}^{\frac{1}{3}}$$

where *n*_1_ is the non-Newtonian index; *f*_1_ is the volume flow rate, cm^3^/s. The total pouring volume was obtained as 804.4895 cm^3^ from Moldflow 2023 software, and the injection time was 0.9872 s. The volume flow rate of the sprue was calculated as *f*_1_ = 814.92 cm^3^/s. Combined with the non-Newtonian index of the material (*n*_1_ = 0.54), the sprue inlet diameter was calculated to be 1.26 cm, which was taken as 1.30 cm in engineering practice.

The empirical formula for calculating the runner diameter is presented in Equation (2):

(2) $$D_{2}=0.172\times {\left(\frac{3n_{2}+1}{n_{2}}f_{2}\right)}^{\frac{1}{3}}$$

where *n*_2_ is the non-Newtonian index; *f*_2_ is the volume flow rate of a single runner, cm^3^/s. The two-gate scheme corresponds to two symmetrical runners, with a volume flow rate *f*_2_ of 407.46 cm^3^/s for each runner. The non-Newtonian index *n*_2_ is determined to be 0.58 from the material data. The calculated runner diameter is 2.14 cm, which is rounded to 2.0 cm for engineering applications.

**Figure 8:**
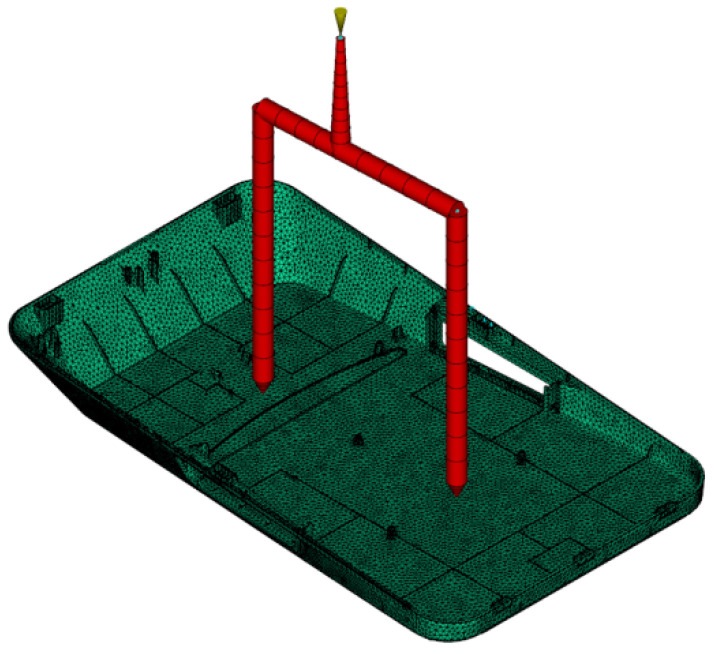
Established hot runner gating system.

A valve-gate type was selected for the gate, with a circular cross-section. The empirical formula for calculating the gate diameter is given in Equation (3):

(3) $$D_{3}=0.0294\times {\left(\frac{3n_{3}+1}{n_{3}}f_{3}\right)}^{\frac{1}{3}}$$

where *n*_3_ is the non-Newtonian index; *f*_3_ is the melt volume flow rate of a single gate, cm^3^/s. The non-Newtonian index *n*_3_ is taken as 0.50 according to the material data. The calculated gate diameter is 0.37 cm, which is adopted as 0.4 cm for engineering applications. The finally established gating system is shown in Figure 8.

#### 2.2.3. Design of Cooling System

The cooling system was designed based on heat transfer theory, with room-temperature cooling water adopted as the cooling medium. The formula for calculating the total heat released by molten plastic solidification during injection molding is expressed as Equation (4):

(4) Q=imΔh

where *Q* is the total heat exchange rate of the mold cavity, kJ/h; *i* is the injection cycle number per unit time, shots/h; *m* is the total mass of plastic per single injection, kg; and Δ*h* is the specific enthalpy drop during plastic solidification, kJ/kg. According to the Moldflow 2023 simulation results, the single-cavity mass of the plastic part is 841.0 g, the mass of the condensate in the hot runner system is 459.0 g, and thus the total mass per single injection *m* = 1300 g. The injection cycle number per hour *i* is 50 shots, and the specific enthalpy drop of PP material during solidification is set to 700 kJ/kg. The total heat exchange rate of the mold is calculated as *Q* = 45,500 kJ/h.

**Figure 9:**
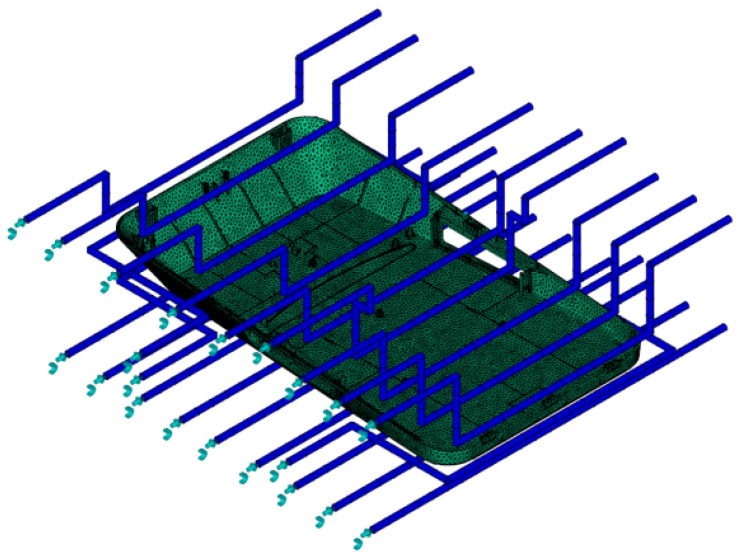
Established cooling system.

Assuming that the heat taken away by the cooling water is balanced with the total heat released by the plastic, the formula for calculating the volume flow rate of cooling water is given by Equation (5):

(5) $$q=\frac{Q}{\rho \cdot c\cdot \Delta T\cdot 60}$$

where *q* is the volume flow rate of cooling water, m^3^/min; *ρ* is the density of water, taken as 1000 kg/m^3^; *c* is the specific heat capacity of water, taken as 4.18 kJ/(kg·°C); and Δ*T* is the temperature difference between the inlet and outlet of cooling water, set to 5 °C. The calculated volume flow rate is 2.177 × 10^−3^ m^3^/min. Combined with the relationship between coolant flow velocity and pipe diameter shown in Table 3, the diameter of the cooling pipeline is determined to be 8 mm when *q* < 5 × 10^−3^ m^3^/min. The finally established cooling system is presented in Figure 9.

### 2.3. Bi-Objective Optimization Method for Injection Molding Process Parameters

#### 2.3.1. Establishment of Response Surface Model Based on Central Composite Design

Response surface methodology (RSM) is an efficient statistical modeling method, which can construct the nonlinear mapping relationship between independent variables and response values through scientific experimental design and greatly reduce the experimental cost [24]. In this study, the Central Composite Design (CCD) method was selected to establish the response surface surrogate model. This method can accurately estimate the linear, quadratic, and interaction effects of independent variables with a small number of experiments, and is suitable for the modeling of multivariable nonlinear processes [25].

Combined with the process characteristics of GF-PP injection molding, six key process parameters, namely melt temperature (*A*), mold temperature (*B*), holding pressure (*C*), holding time (*D*), injection time (*E*) and cooling time (*F*), were selected as the experimental independent variables, and the maximum warpage deformation and maximum residual stress of the plastic part were taken as the optimization response indexes. Combined with the process range recommended by the material manufacturer, the level values of each independent variable were determined, as shown in Table 4.

A second-order multiple regression model widely used in engineering was adopted to construct the response surface surrogate model, and its general expression is shown as Equation (6):

(6) $$Y=\beta_{0}+\sum_{i=1}^{k}\beta_{i}x_{i}+\sum_{i=1}^{k}\beta_{ii}x_{i^{2}}+\sum_{i<j}^{k}\beta_{ij}x_{i}x_{j}+\varepsilon$$

where *Y* is the response value (warpage deformation/residual stress); *β*_0_ is the constant term; *β_i_*, *β_ii_* and *β_ij_* are the regression coefficients of the linear term, quadratic term and interaction term, respectively; *x_i_* and *x_j_* are the independent variables; *k* is the number of independent variables; and *ε* is the random error.

During the model construction, analysis of variance (ANOVA) was performed to conduct the significance test of model terms, and the insignificant model terms were eliminated with *p* > 0.05 as the threshold. The fitting accuracy of the model was evaluated by the coefficient of determination *R*^2^ and the adjusted coefficient of determination *R*_adj_^2^ The reliability verification of the model was completed through the normal distribution test of residuals, fitting analysis of predicted values versus actual values, and distribution analysis of residuals versus predicted values. Finally, high-precision second-order response surface models of warpage deformation, residual stress and key process parameters were established, respectively. Taking the verified second-order response surface models as the objective functions and the engineering value range of each process parameter as the constraint conditions, a bi-objective optimization mathematical model focusing on minimizing warpage deformation and residual stress was established.

#### 2.3.2. Solution of the Bi-Objective Optimization Model Based on NSGA-II

Aiming at the two mutually conflicting optimization objectives of warpage deformation and residual stress, the Non-Dominated Sorting Genetic Algorithm II (NSGA-II) was introduced to solve the Pareto optimal solution set [26]. By adopting non-dominated sorting, crowding distance calculation and elitist preservation strategies, this algorithm can efficiently solve the global optimal solution set of multi-objective optimization problems, and has better convergence and distribution uniformity than traditional Genetic Algorithms [13].

**Figure 10:**
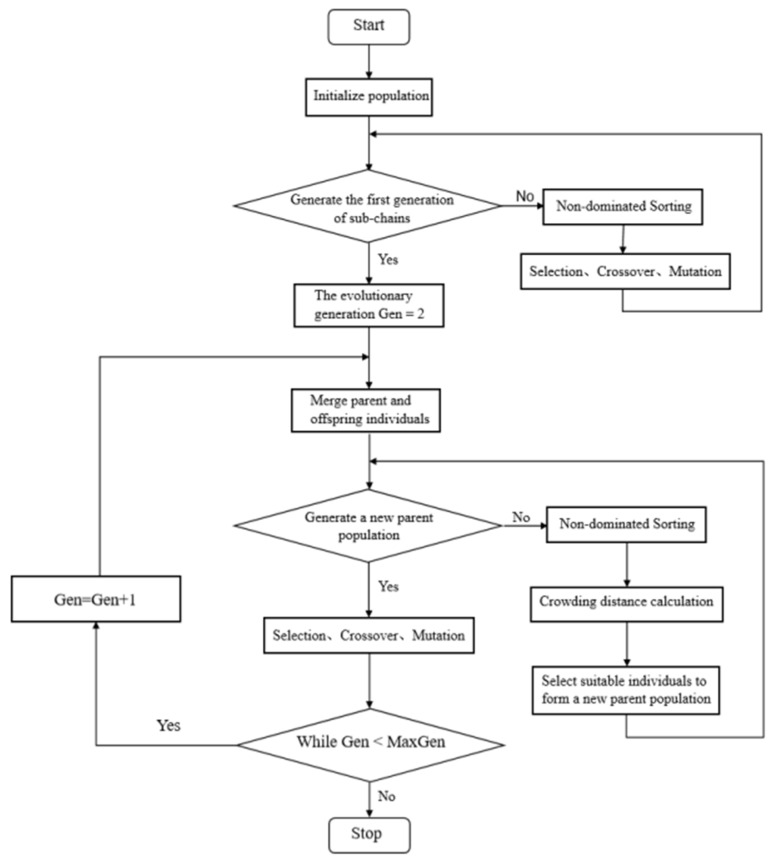
Flow chart of the NSGA-II optimization algorithm.

The solution program of the NSGA-II algorithm was implemented using Matlab R2023a software. Combined with the scale and characteristics of the optimization problem, the core operating parameters of the algorithm were set as follows: population size of 50, maximum iteration number of 300 generations, crossover probability of 0.8, and mutation probability of 0.02. The algorithm flow is shown in Figure 10. Firstly, the population was initialized, and the first-generation offspring population was generated through selection, crossover and mutation operations. If the individuals failed to meet the constraint conditions, non-dominated sorting was performed. Subsequently, the evolution generation was set as Gen = 2, the parent and offspring populations were merged, and a new generation of parent population was generated by screening according to non-dominated sorting. Then, selection, crossover, and mutation operations were performed on the new parent population to generate new offspring individuals. This process was iterated continuously: if the current generation was less than the maximum generation, the generation number was increased, and the optimization was continued; otherwise, the algorithm was terminated, and the final optimization results were output. The Pareto optimal solution set for bi-objective optimization was obtained through an iterative solution. Combined with the molding quality requirements of plastic parts, the optimal combination of process parameters that balanced the dual quality objectives was screened out, and the optimization results were verified through Moldflow 2023 numerical simulation.

### 2.4. Co-Simulation Method for Mechanical Properties of Plastic Parts Considering Molding History

#### 2.4.1. Theoretical Basis of Anisotropic Mechanics for Fiber-Reinforced Composites

Glass fiber-reinforced polypropylene melt is a typical non-Newtonian fluid whose shear viscosity dependence on shear rate and temperature is described by the Cross–WLF model [27], consistent with the viscosity model used in the aforementioned Moldflow injection molding simulation. The expression is given in Equation (7):

(7) $$\eta \left(\dot{\gamma },T,p\right)=\frac{\eta_{0}\left(T,p\right)}{1+{\left(\eta_{0}\dot{\gamma }/\tau^{*}\right)}^{1-n}}$$

where *η* is the melt shear viscosity, Pa·s; *η*_0_ is the zero-shear viscosity, Pa·s; $\dot{\gamma }$ is the shear rate, s^−1^; τ^*^ is the critical shear stress, Pa; and *n* is the non-Newtonian index. The zero-shear viscosity *η*_0_ is calculated by the WLF equation, which accounts for the effect of melt temperature on viscosity.

Aiming at the fiber orientation characteristics of glass fiber-reinforced composites, the second-order orientation tensor was adopted to characterize the spatial distribution state of fibers, as shown in Equation (8). This method overcomes the disadvantages of cumbersome calculation and low efficiency of the probability distribution function, and can accurately map the influence of fiber orientation on the anisotropy of material mechanical properties.

(8) $$A_{ij}=\oint p_{i}p_{j}\phi \left(p\right)dp$$

where *A_ij_* is the second-order fiber orientation tensor, which is a symmetric matrix; *p_i_* and *p_j_* are the projections of the fiber unit direction vector ***p*** on the coordinate axes; and *ϕ*(***p***) is the probability distribution function of fiber orientation. When the diagonal component of the tensor *a*_11_ = 1, the fibers are completely oriented along a single direction; when all three diagonal components are equal to 1/3, the fibers present a random spatial distribution, and the material exhibits isotropic properties. In this paper, the maximum eigenvalue of the fiber orientation tensor was used to characterize the fiber orientation degree; the closer the eigenvalue is to 1, the higher the fiber orientation degree.

#### 2.4.2. Data Mapping Method for Moldflow-Ansys Co-Simulation

**Figure 11:**
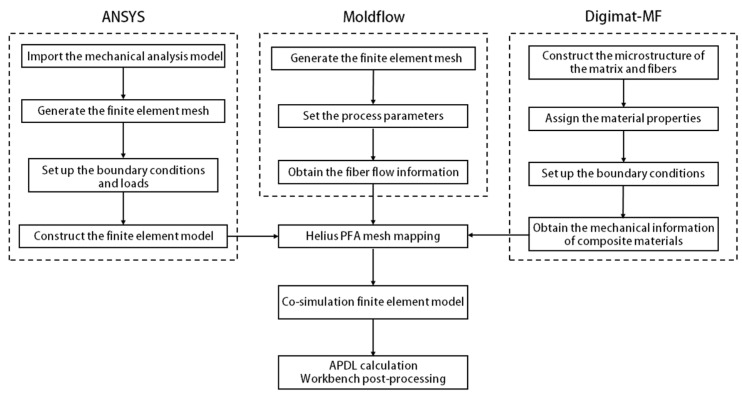
Co-simulation flowchart.

**Figure 12:**
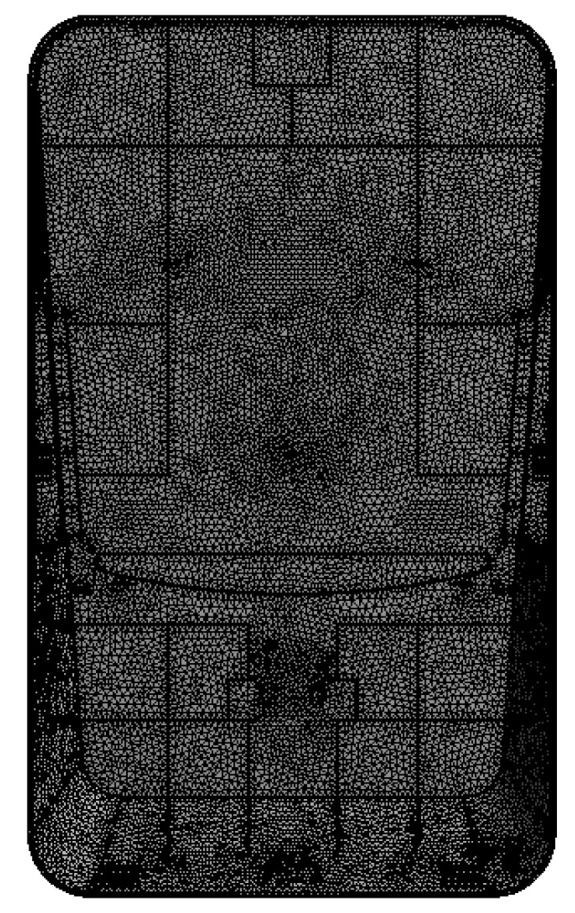
Meshing model in Workbench software.

Traditional mechanical simulation regards plastic parts as isotropic homogeneous materials, which fails to consider the effects of injection molding-induced defects such as fiber orientation, residual stress and weld lines on mechanical properties, resulting in a large deviation between predicted results and actual service behavior. In this paper, Autodesk Helius PFA 2023 software was adopted to realize bidirectional data mapping between injection molding information and structural mechanical simulation. The entire multi-software co-simulation workflow is shown in Figure 11, and the complete process is as follows:

(1) Based on the simulation results of the optimal process by Moldflow 2023 described above, the result file in .sdy format containing fiber orientation tensor, residual stress distribution, and weld line positions was extracted.
(2) A high-precision structural mesh matching the Moldflow mesh was established in Ansys Workbench 2023 R2, and the mesh element size was uniformly set equivalent to the wall thickness of the plastic part (3 mm), as shown in Figure 12, to ensure the accurate mapping of molding information, and the mesh file in .inp format was output.
(3) The above two types of files were imported into the Advanced Material Exchange module of Helius PFA 2023, and mesh matching was completed by combining automatic alignment with interactive fine-tuning to guarantee the mapping accuracy of molding information at Gaussian points.
(4) After the mapping of fiber orientation, residual stress and weld line information was completed, files in .inp, .hin and .sif formats containing anisotropic material properties were exported. Mechanical calculations were performed using the Mechanical APDL solver, and finally, the stress and strain results were extracted in Workbench.

#### 2.4.3. Boundary Conditions and Load Settings for Mechanical Property Analysis

**Figure 13:**
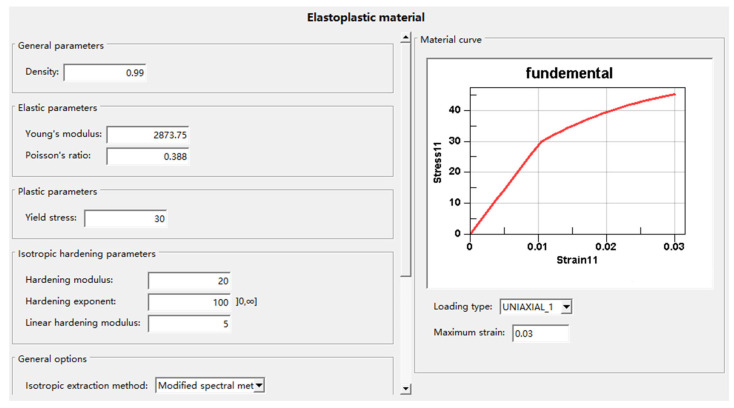
Parameter setting of the matrix material.

The polypropylene/30% short glass fiber (PP/30% GF) composite is a typical anisotropic elastoplastic material, and its equivalent mechanical property parameters cannot be directly obtained in ANSYS 2023 R2 software. Therefore, the Mean Field (MF) module of Digimat 2023.1 software was adopted to establish a meso-mechanical model of the composite, and the full mechanical property parameters required for the simulation were calculated. In this model, polypropylene (PP) was used as the matrix phase and short glass fibers as the reinforcement phase to construct the Representative Volume Element (RVE) of the composite. The glass fiber reinforcement phase adopted a linear elastic constitutive model with the input parameters: Young’s modulus of 70,000 MPa and Poisson’s ratio of 0.3. The PP matrix phase adopted an elastoplastic constitutive model, and its basic physical parameters were retrieved from the material library of Moldflow 2023 software, including a density of 0.99 g/cm^3^, Young’s modulus of 2873.75 MPa, and Poisson’s ratio of 0.38. Combined with the mechanical property test data of the material and the simulation convergence requirements, the matrix yield stress was set to 30 MPa, the hardening modulus to 20 MPa, the hardening exponent to 100, and the linear hardening exponent to 5. The parameter setting interface of the matrix material is shown in Figure 13.

**Figure 14:**
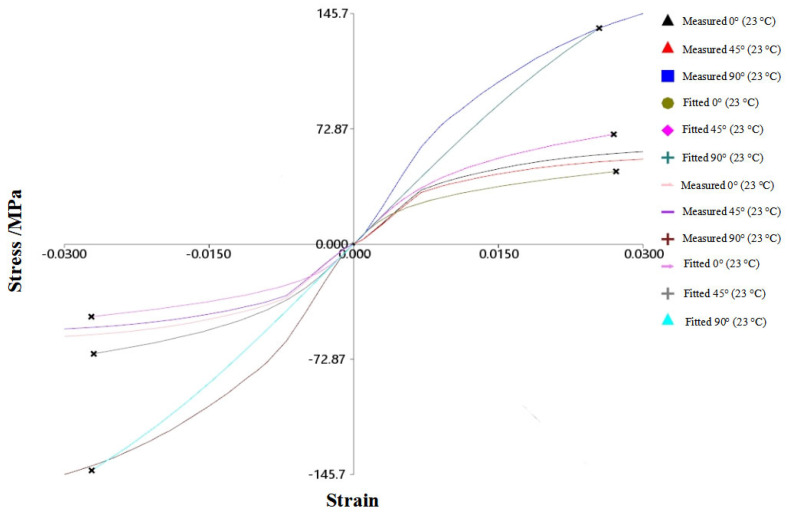
Stress–strain curves of the composite at different orientations.

Short fiber-reinforced composites fabricated by injection molding exhibit significant anisotropy in mechanical properties, and fiber orientation directly determines the load-bearing capacity of the component in different directions. To accurately predict the mechanical response of the face shell under actual loading conditions, it is necessary to clarify the stress–strain relationships of the material at three typical fiber orientation angles of 0°, 45° and 90°. In the simulation, the aspect ratio of glass fibers was set to 25, and the stress–strain curves of the composite at each orientation calculated by the Digimat-MF module are shown in Figure 14, which provides accurate material property inputs for the subsequent co-simulation.

**Figure 15:**
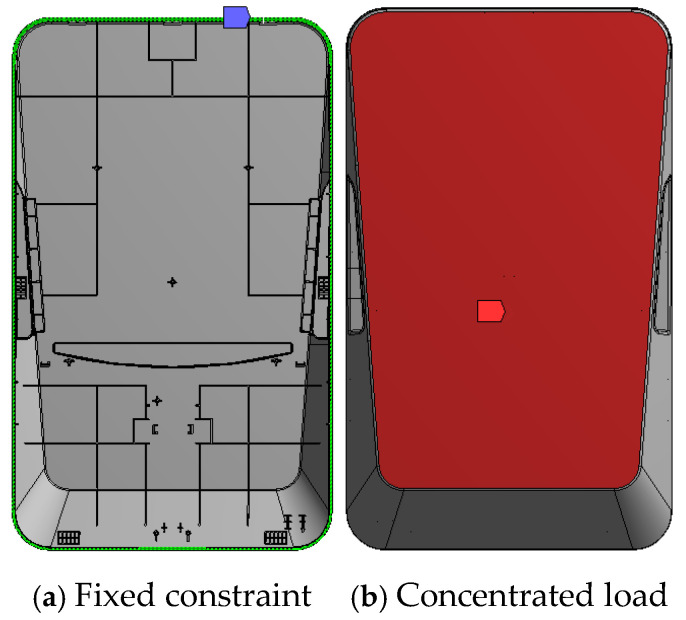
Boundary conditions.

The settings of boundary conditions and loads fully matched the actual assembly relationship and service conditions of the fan face shell. A fixed constraint with all degrees of freedom was applied to the edge of the mounting frame on the back of the fan face shell (as shown in Figure 15a) to simulate the actual assembly constraint state of the face shell. Referring to the mechanical property test standards for similar industrial fan shells, a concentrated load of 150 N, perpendicular to the surface of the face shell and acting inward, was applied to the central area of the front surface of the face shell (as shown in Figure 15b).

### 2.5. Injection Molding Experimental Equipment and Mold Trial Scheme

The injection molding machine used for mold trial production was the TTI-1000SeII model manufactured by Donghua Machinery Co., Ltd. (Dongguan, China), with a clamping force of 1000 T. Its main parameters are listed in Table 5.

After the mold installation was completed and debugged until the melt filling was stable, the optimized process parameters were adopted for mold trial production. Ten qualified plastic parts were randomly selected, and the warpage deformation was measured by a SEREIN coordinate measuring machine (CMM) (Shenzhen, China). The measurement environment was controlled at a temperature of (20 ± 2) °C and a relative humidity of 50% ± 5%, with the instrument measurement accuracy of MPE_E ≤ 2.5 + L/300 μm. The mounting frame and central load-bearing area of the plastic part were selected as the measurement regions, and a total of 15 measurement points were set. Each point was repeatedly measured three times, and the average value was taken. Finally, the measured values of the maximum warpage deformation of the 8 samples were calculated and compared to the algorithm prediction results to verify the engineering accuracy of the optimization method.

## 3. Results and Discussion

### 3.1. Analysis of Initial Simulation Results

**Figure 16:**
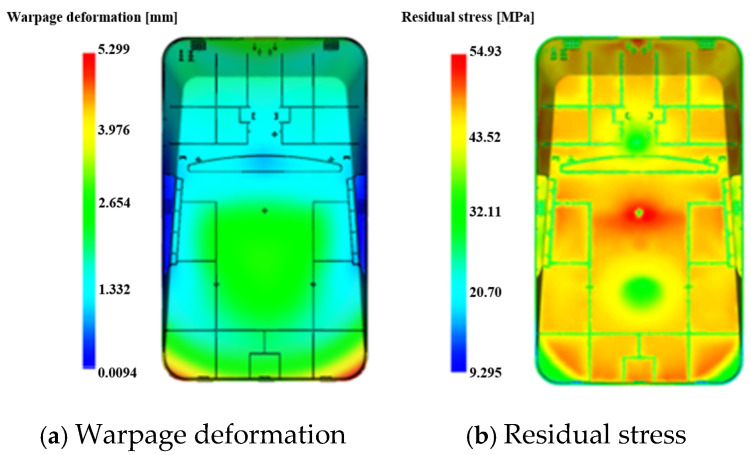
Contour plots of molding quality under initial process parameters.

Initial simulations were performed based on the established numerical model. The melt temperature and mold temperature were recommended by the material manufacturer as 230 °C and 40 °C, respectively. With these two parameters, the injection time corresponding to the optimal molding quality was determined to be 0.98 s by querying the Molding Window sequence in Moldflow software. The holding pressure was set to 55 MPa, which accounted for 70% of the default filling pressure; the holding time was set to the default value of 10 s, and the cooling time was set to 20 s. The analysis results of warpage and residual stress are shown in Figure 16. The maximum warpage deformation was 5.299 mm, and the maximum residual stress was 54.93 MPa. According to product assembly requirements, the warpage value must be less than 2.5 mm, and excessive residual stress should be avoided simultaneously. Therefore, the initial process fails to meet the production requirements and needs further optimization.

### 3.2. Significance and Accuracy Verification Results of the Response Surface Model

The maximum warpage deformation (mm) and maximum residual stress (MPa) of the molded plastic parts were taken as the response indices to characterize the injection molding quality of the plastic parts. A CCD scheme containing 86 groups of experiments was generated using Design-Expert 13.0 software. The full-process numerical simulation of injection molding for all experimental groups was completed via Moldflow 2023 software. To reduce the length of the main text while retaining the representativeness of the experimental data, a subset of 15 representative runs is presented in Table 6, including five center point replicates (for verifying experimental repeatability), five axial point runs (for estimating quadratic effects of process parameters), and five factorial point runs (for estimating linear and interaction effects). The complete 86-run CCD experimental dataset is provided in Table S1 in the Supplementary Materials to ensure the transparency and reproducibility of this study.

Based on the CCD experimental dataset, second-order response surface models for warpage deformation and residual stress were established, respectively. The cooling time (*F*) exerted no significant effect on both warpage deformation and residual stress (*p* > 0.9) and was thus eliminated from the models. The results of the analysis of variance (ANOVA) are presented in Table 7 and Table 8, respectively.

The ANOVA results indicate that the F-values of the response surface models for warpage deformation and residual stress are 443.92 and 265.99, respectively, with all *p*-values less than 0.0001. The regression effects of both models reach an extremely significant level, and the probability that model variation is caused by random errors is less than 0.01%, indicating strong statistical significance. Further fitting accuracy verification shows that the coefficient of determination *R*^2^ and adjusted coefficient of determination *R*_adj_^2^ of the warpage deformation model are 0.9965 and 0.9942, respectively; while those of the residual stress model are 0.9947 and 0.9909, respectively. The *R*^2^ values of both models are close to 1, which means the models can explain more than 99% of the variation in response values and have extremely high fitting accuracy for the nonlinear mapping relationship between process parameters and molding quality objectives. The simplified mathematical models are presented in Equation (9) and Equation (10), where *W* and *R* represent the warpage deformation value and residual stress value, respectively.

(9) $$\begin{array}{ll} W= & -61.91204+0.550787A+0.593651B+1.01949C+0.000842D-1.9811E\\ & -0.00355AB-0.009288AC-0.000284AD+0.012833AE+0.000602BC\\ & -0.005647BD-0.002455BE-0.00071CD-0.0011691CE+0.03192DE\\ & -0.001088A^{2}-0.002431B^{2}+0.000222C^{2}+0.003706D^{2} \end{array}$$

(10) $$\begin{array}{ll} R= & 210.12039-1.42396A+9.86549B-9.90162C-8.42868D+49.37617E\\ & -0.076568AB+0.077444AC+0.076433AD-0.37925AE+0.01554BC\\ & -0.000557BD-0.21BE+0.005773CD-0.0278DE+0.02953A^{2}-0.029895B^{2}\\ & +0.009781C^{2}-0.004211D^{2}-0.51499E^{2} \end{array}$$

**Figure 17:**
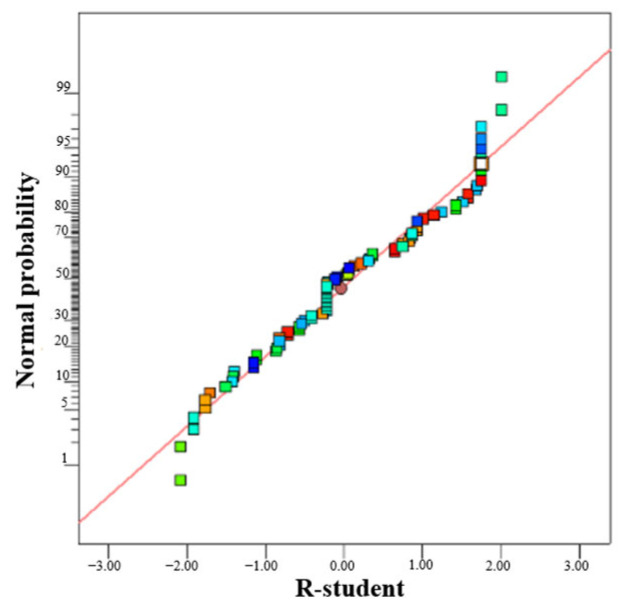
Normal probability plot of residuals for *W*.

**Figure 18:**
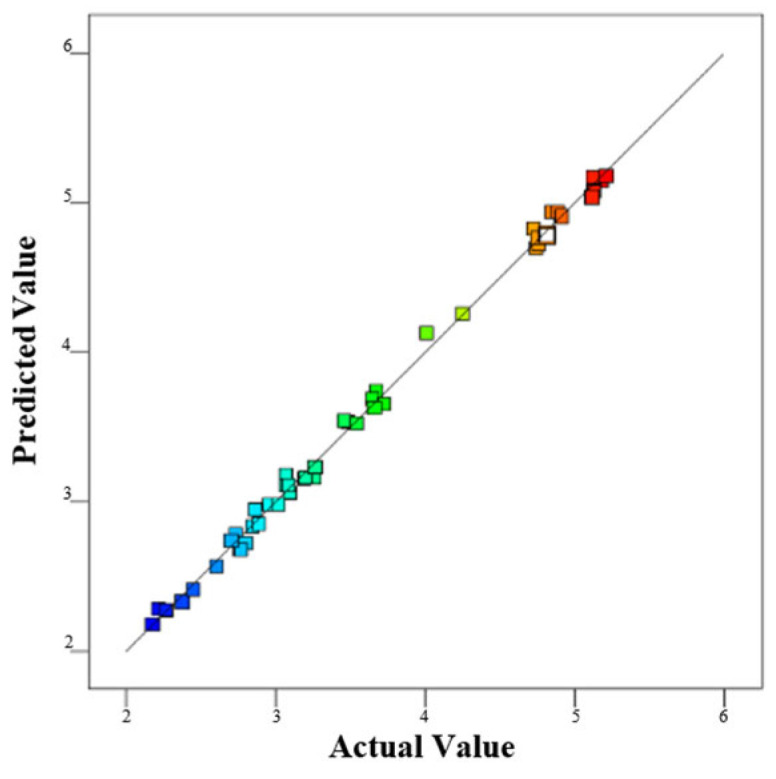
Predicted versus actual values for *W*.

**Figure 19:**
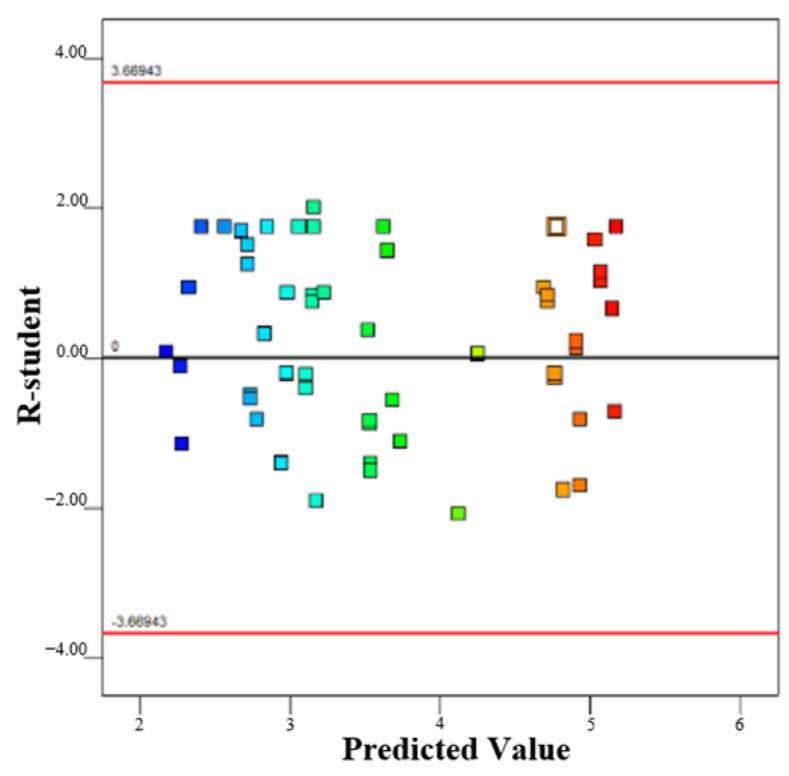
Residuals versus predicted values for *W*.

**Figure 20:**
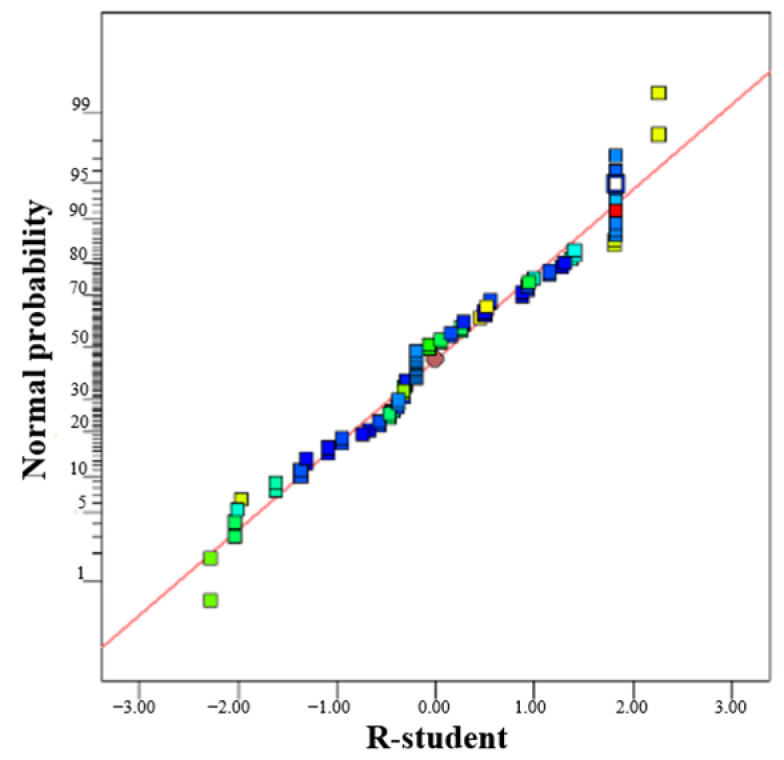
Normal probability plot of residuals for *R*.

**Figure 21:**
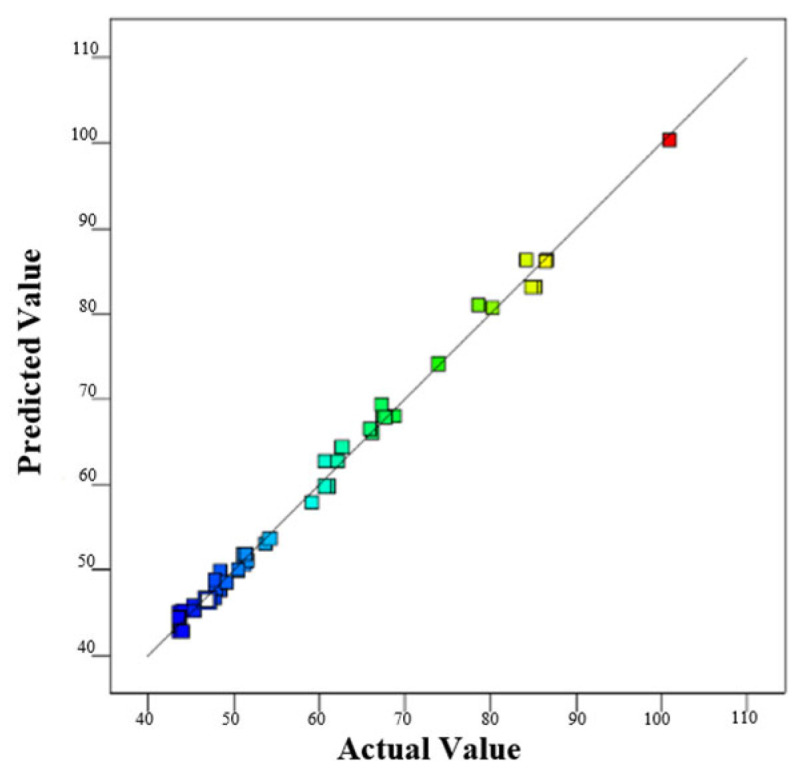
Predicted versus actual values for *R*.

**Figure 22:**
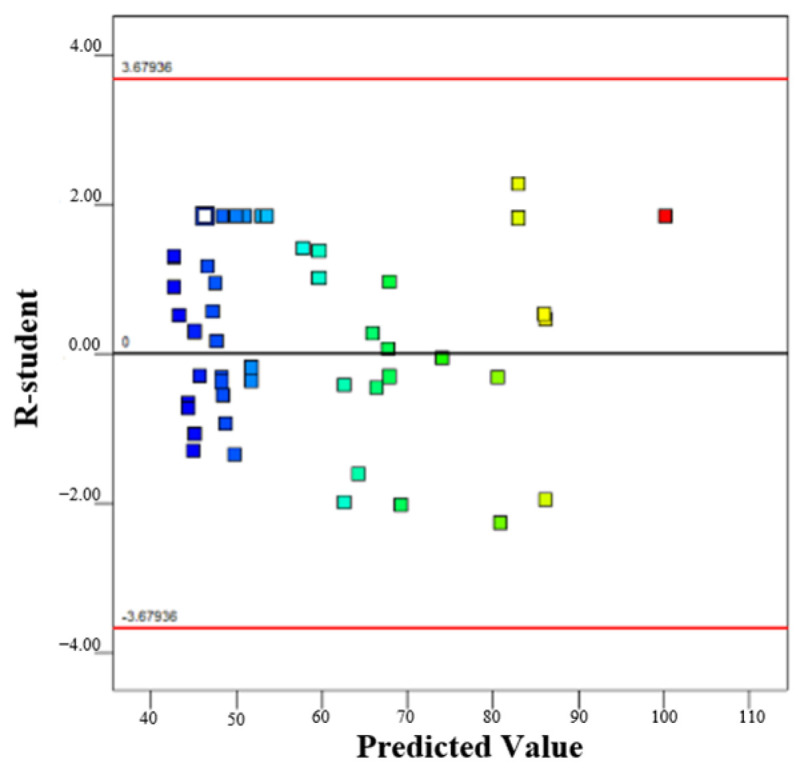
Residuals versus predicted values for *W*.

To further verify the accuracy of the simplified models, the normal probability plot of residuals, the predicted versus actual values plot, and the residuals versus predicted values plot were adopted for accuracy validation, as shown in Figure 17, Figure 18, Figure 19, Figure 20, Figure 21 and Figure 22.

The reliability verification of the models was completed using the above three diagnostic plots. The results show that the residual points of both the warpage deformation and residual stress models are uniformly distributed along the normal distribution line without obvious deviation. The predicted values of the models are almost coincident with the actual simulation values from Moldflow along the 45° reference line, with no significant dispersion. There is no obvious regularity or abnormal trend in the distribution of residuals with the predicted values. The above results confirm that the established second-order response surface models have no lack of fit, and the residuals conform to the normal distribution assumption. The models exhibit excellent prediction performance and reliability, and can be applied to the subsequent multi-objective optimization of injection molding process parameters. In addition, the five repeated experiments at the center point (*A* = 230 °C, *B* = 40 °C, *C* = 50 MPa, *D* = 20 s, *E* = 2 s, *F* = 20 s) yielded consistent warpage deformation of 3.088 mm and residual stress of 51.51 MPa, indicating excellent repeatability of the numerical simulation experiments. As shown in Table 9, the 95% prediction intervals (PI) for the center point responses calculated by Design-Expert 13.0 are [2.958, 3.249] mm for warpage deformation and [48.974, 54.545] MPa for residual stress. The observed values fall completely within the prediction intervals, with relative errors of only 0.484% and 0.483%, respectively, further confirming the high fitting accuracy of the models.

### 3.3. Pareto Solution Set and Optimal Process Scheme for Bi-Objective Optimization

Taking the verified second-order response surface models as the objective functions and the engineering value ranges of each process parameter as the constraint conditions, a bi-objective optimization mathematical model focusing on minimizing warpage deformation and residual stress was established. The expression of the optimization model is shown in Equation (11):

(11) $$\left\{\begin{array}{l} F=\min\left[W,R\right]\\ s.t.\left\{\begin{array}{c} 200\leq A\leq 260\\ 20\leq B\leq 60\\ 20\leq C\leq 80\\ 10\leq D\leq 30\\ 1\leq E\leq 3 \end{array}\right. \end{array}\right.$$

**Figure 23:**
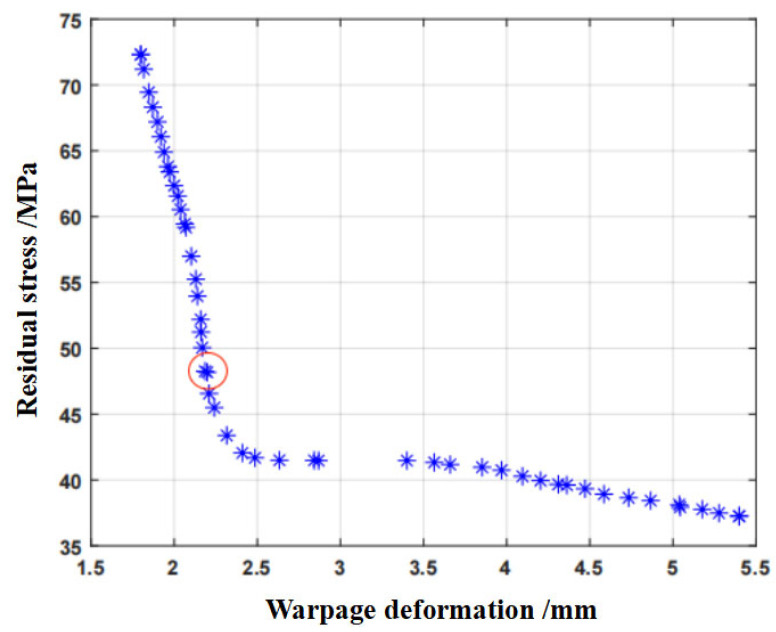
Pareto optimal solution set.

The Pareto optimal solution set for bi-objective optimization was solved based on the NSGA-II algorithm, which contains 50 groups of non-dominated optimal solutions, as shown in Figure 23. The optimal range of warpage deformation is 1.5~5.5 mm, and the optimal range of residual stress is 35~75 MPa. The Pareto front clearly presents the conflicting relationship between the two optimization objectives: the residual stress of the plastic part shows a significant upward trend with the decrease in warpage deformation, which further verifies the necessity of bi-objective collaborative optimization. Combined with the engineering application requirements of the fan face shell plastic part (warpage deformation ≤ 2.5 mm) and considering the structural mechanical properties of the plastic part (avoiding excessive residual stress), the optimal combination of process parameters balancing the dual quality objectives was screened from the Pareto front: melt temperature of 259.99 °C, mold temperature of 21.98 °C, holding pressure of 55.07 MPa, holding time of 30.0 s, and injection time of 3.0 s. The optimized predicted values corresponding to this scheme are warpage deformation of 2.199 mm and residual stress of 48.178 MPa, which not only meet the molding accuracy requirements of the plastic part, but also control the residual stress within a reasonable range.

### 3.4. Simulation Verification

**Figure 24:**
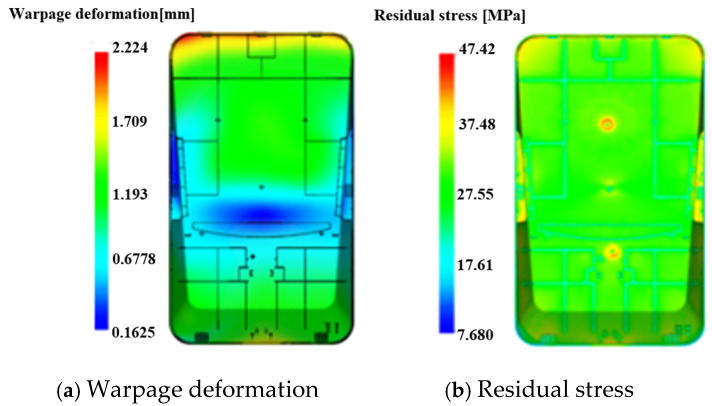
Contour plots of molding quality with optimized process parameters.

The optimal process parameters obtained from the optimization were substituted into Moldflow software for simulation verification, with the cooling time set to 20 s. The results of warpage deformation and residual stress are shown in Figure 24. The simulated warpage deformation and residual stress were 2.224 mm and 47.420 MPa, respectively, with relative errors of 1.124% and 1.598% compared to the predicted values by the NSGA-II algorithm, indicating high prediction accuracy.

In contrast, the simulation results under the initial process parameters recommended by the material manufacturer and software were 5.299 mm for warpage deformation and 54.93 MPa for residual stress. After algorithm optimization, the warpage deformation was reduced by 58.030%, and the residual stress was decreased by 13.671%. Both core molding quality indexes were significantly improved, especially the warpage deformation, which was greatly suppressed, fully meeting the molding accuracy requirements of the plastic parts.

### 3.5. Analysis of Microstructure and Mechanical Behavior of Plastic Parts

#### 3.5.1. Fiber Orientation Distribution Characteristics of Plastic Parts

**Figure 25:**
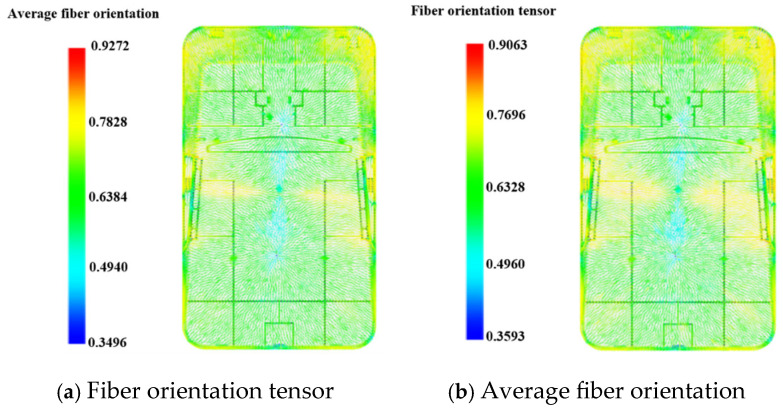
Fiber orientation diagrams.

The fiber orientation distribution of the plastic part under the optimal process is presented in Figure 25. The maximum eigenvalue of the average fiber orientation tensor of the plastic part is 0.7. Specifically, the fiber orientation degree in the melt flow end region far from the gate reaches up to 0.9272, while the fiber orientation degree is relatively low and unevenly distributed in the regions near the gate, ribs and stiffeners. This distribution characteristic is directly associated with the design of the dual-gate gating system described previously. The dual-gate configuration achieves balanced melt filling. The melt flow path is stable in the region away from the gate, enabling fibers to be fully oriented along the flow direction. In contrast, intense fiber interaction occurs near the gate due to melt impingement and runner direction change. Meanwhile, the abrupt variation in flow resistance at ribs and stiffeners results in disordered fiber orientation, which is the core cause for the frequent occurrence of tiny shrinkage marks in these regions. Overall, the combination of the dual-gate configuration and optimal process parameters realizes uniform fiber orientation in the main area of the plastic part, which provides a microstructural foundation for improving the macroscopic mechanical properties of the plastic part.

#### 3.5.2. Anisotropy of Tensile Modulus Induced by Fiber Orientation

**Figure 26:**
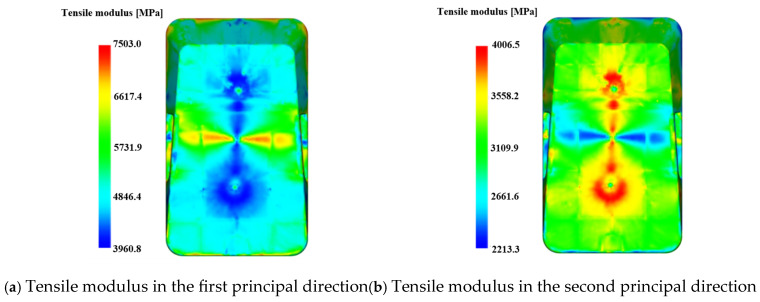
Contour plots of tensile modulus.

The distribution of tensile modulus in the first and second principal directions of the plastic part under the optimal process is shown in Figure 26. The first principal direction coincides with the melt flow direction, with an average tensile modulus of approximately 6500 MPa and a maximum value of 7503 MPa. The second principal direction is perpendicular to the main melt flow direction, with an average tensile modulus of about 3500 MPa and a minimum value of only 2213 MPa. The average tensile modulus in the first principal direction is 1.85 times that in the second principal direction, with a maximum difference of 3.4 times, which clearly demonstrates the significant mechanical anisotropy of glass fiber-reinforced polypropylene injection-molded parts. Moreover, the distribution law of tensile modulus is completely consistent with the fiber orientation distribution. The results confirm that the high orientation of fibers along the melt flow direction significantly improves the material stiffness in this direction. The process parameters optimized previously, such as holding pressure and holding time, further enhance the fiber orientation in the main flow direction by stabilizing the melt filling and compaction processes, thus achieving the stiffness improvement in the load-bearing direction of the plastic part.

#### 3.5.3. Comparison of Mechanical Behaviors Between Co-Simulation and Traditional Simulation

**Figure 27:**
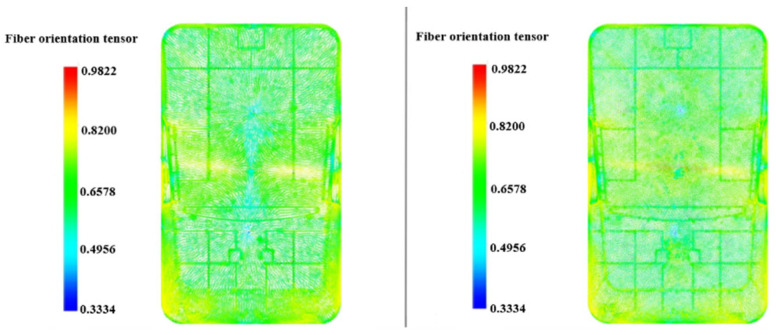
Mapping results of the fiber orientation tensor.

**Figure 28:**
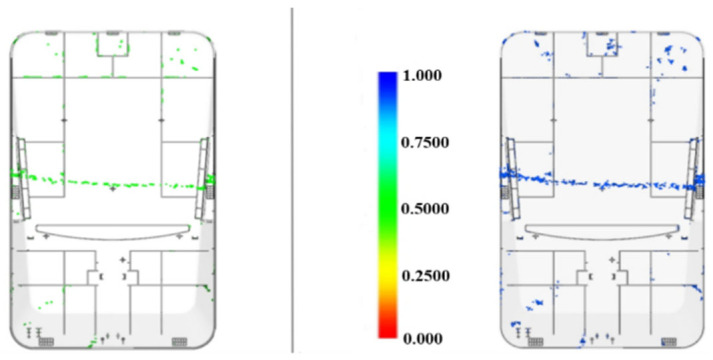
Mapping results of the weld surface.

The mapping results of the fiber orientation tensor and weld surface are shown in Figure 27 and Figure 28. The orientation tensor obtained from mold flow analysis (left) exhibits good consistency after being mapped to the structural analysis model (right), and the weld surface results also accurately inherit the distribution of weld surfaces in the plastic part from the original mold flow analysis.

**Figure 29:**
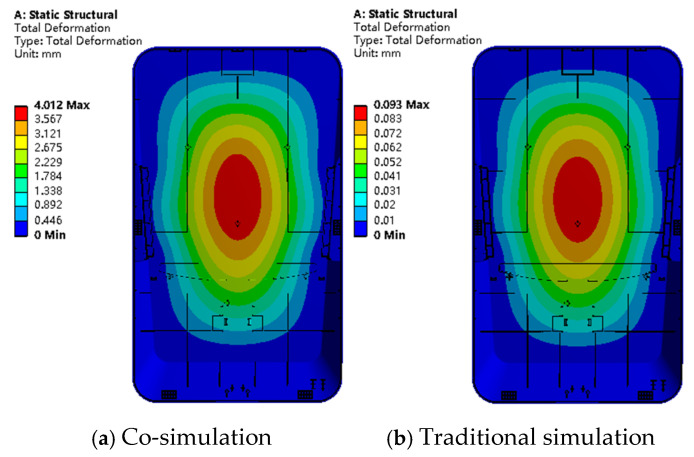
Comparison of total deformation between co-simulation and traditional simulation.

**Figure 30:**
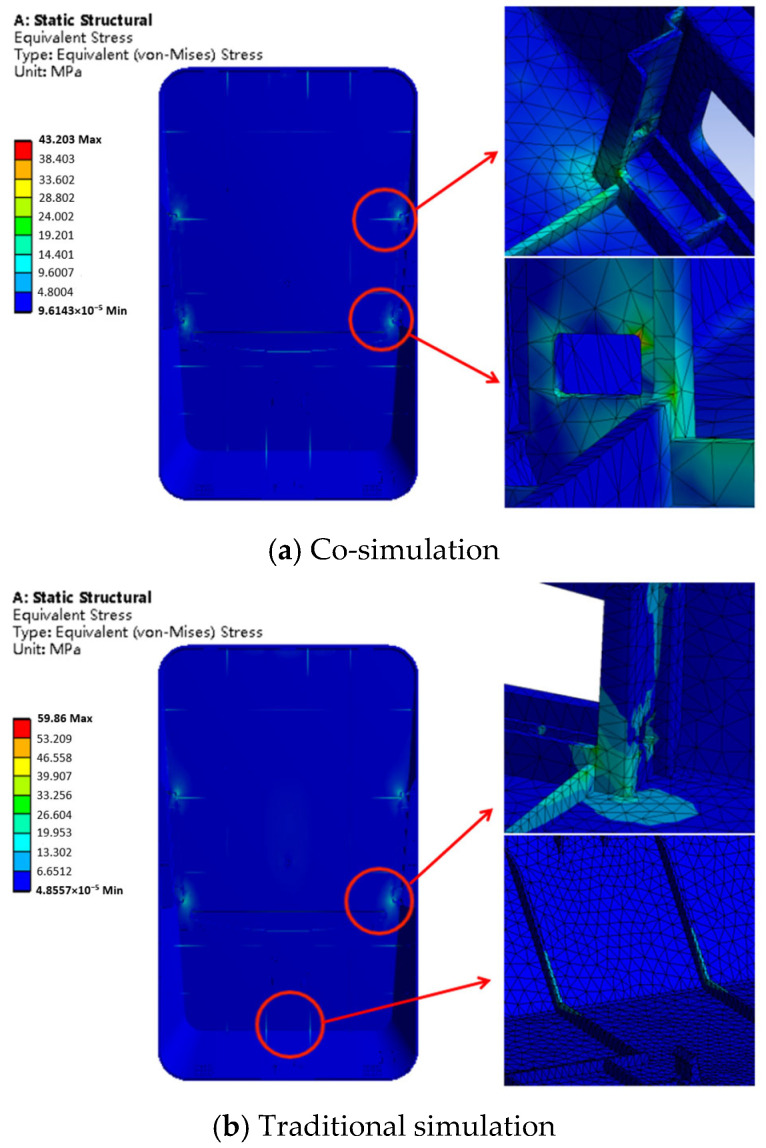
Comparison of equivalent stress between co-simulation and traditional simulation.

The co-simulation considering molding history and the traditional isotropic simulation were respectively adopted to calculate the mechanical behavior of the plastic part under rated load, and the comparison results of total deformation and equivalent stress are presented in Figure 29 and Figure 30.

Deformation results demonstrate that the maximum deformation of the molded part predicted by conventional isotropic simulations is only 0.09 mm, while that predicted by the co-simulation reaches 4.01 mm, a significant difference between the two methods. The core reason is that the traditional simulation ignores the anisotropy induced by fiber orientation and assumes uniform material stiffness in all directions, which seriously overestimates the deformation resistance of the plastic part perpendicular to the fiber direction and makes the deformation prediction results lack engineering reference value. In contrast, the co-simulation completely retains the fiber orientation information from injection molding, accurately reflects the stiffness difference in the material in different directions, and the deformation results are more consistent with the actual service behavior of the plastic part.

The stress results indicate that the stress concentration regions predicted by the two simulation methods are consistent, which are all located at the frame skeletons on both sides and the back stiffeners of the plastic part. However, the maximum equivalent stress predicted by the co-simulation is 43.20 MPa, which is significantly lower than 59.86 MPa from the traditional simulation. This is because the co-simulation takes into account the regulatory effect of fiber orientation on stress distribution. The high stiffness along the fiber direction realizes uniform stress dispersion and effectively alleviates local stress concentration, while the traditional simulation cannot reflect this effect, resulting in conservative stress prediction results.

According to the co-simulation results, the fan face shell manufactured under the optimal process has a maximum deformation of 4.01 mm under an extreme load of 150 N, which is far lower than the maximum allowable deformation of 10 mm for the product. The maximum equivalent stress is 43.20 MPa, which is only 38.8% of the material fracture stress (111.2 MPa), indicating a sufficient safety margin.

### 3.6. Mold Trial Verification

**Figure 31:**
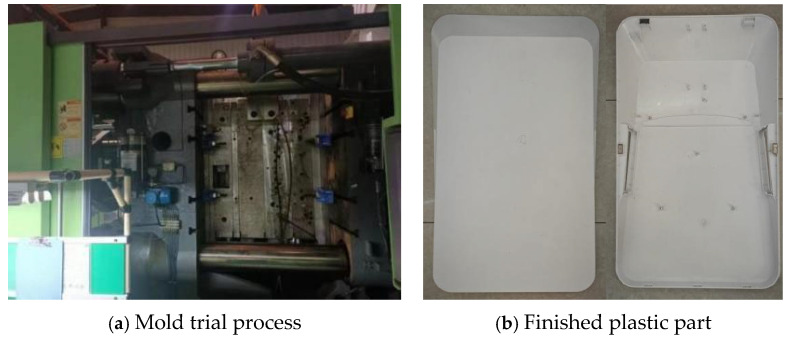
Mold trial process and finished product.

After the mold was installed and debugged to achieve stable melt filling, the optimized process parameter combination was adopted for mold trial production: melt temperature of 259.99 °C (rounded to 260.0 °C), mold temperature of 21.98 °C (rounded to 22.0 °C), holding pressure of 55.07 MPa (rounded to 55.0 MPa), holding time of 30.0 s, and injection time of 3.0 s. The mold trial process and the finished plastic parts are shown in Figure 31.

**Figure 32:**
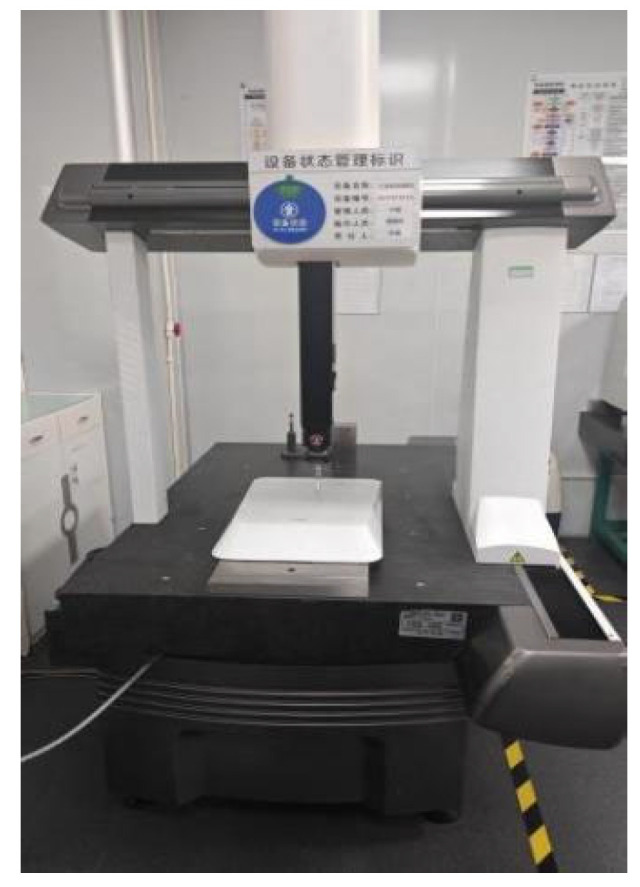
Warpage measurement using a coordinate measuring machine.

A coordinate measuring machine (CMM) was employed to measure the warpage deformation of 10 randomly selected plastic parts. The measurement process is illustrated in Figure 32, and the corresponding results are summarized in Table 10. The average maximum warpage deformation of the 8 samples is 2.044 mm, with a relative error of 7.583% compared to the algorithm-predicted value. The error is within the engineering acceptable range, which verifies the engineering accuracy and applicability of the process optimization method proposed in this study.

## 4. Conclusions

To address the critical challenge of molding quality control for large-size glass fiber-reinforced polypropylene (GF-PP) fan face shells, this study proposes a systematic research methodology integrating RSM-NSGA-II bi-objective process optimization, meso-macro coupled molding-structure co-simulation, and full-chain engineering verification. Compared with the conventional RSM-NSGA-II process optimization framework, this work is the first to apply this hybrid methodological system to large flat GF-PP shells with dimensions exceeding 500 mm, effectively solving the strongly conflicting collaborative optimization problem between warpage deformation and residual stress. Furthermore, a complete correlation chain of “process parameter optimization—microscopic fiber orientation distribution—macroscopic mechanical properties” is established, enabling accurate quantitative characterization of the mechanical anisotropy induced by fiber orientation. Taking a 30% GF-PP fan face shell as the research object, this paper systematically investigates the bi-objective optimization of the injection molding process, mechanical property co-simulation incorporating molding history, and mold trial verification. The main conclusions are drawn as follows:

(1) The gating system optimization results for the large-scale flat fan face shell demonstrate that the dual-gate configuration achieves balanced melt filling, with a filling time of 2.629 s and a flow front temperature drop of 4.7 °C, which is within the reasonable control range of 2–5 °C. Moreover, the weld line defects on the outer surface of the plastic part are significantly fewer than those of the triple-gate and quadruple-gate schemes, which effectively ensures the appearance quality and molding uniformity of the plastic part and lays a foundation for the subsequent optimization of molding quality.
(2) The second-order response surface models of warpage deformation and residual stress established based on CCD experiments reach an extremely significant level (*p* < 0.0001). The coefficient of determination *R*^2^ of the warpage deformation model is 0.9965, and that of the residual stress model is 0.9947. Both models can explain more than 99% of the variation in response values, and have extremely high fitting accuracy and prediction reliability for the nonlinear mapping relationship between process parameters and molding quality indexes. The Pareto optimal solution set for bi-objective optimization is obtained by the NSGA-II algorithm. Combined with the engineering assembly requirements (warpage deformation ≤ 2.5 mm) and mechanical performance control requirements of the plastic part, the optimal combination of process parameters is screened out: melt temperature of 260 °C, mold temperature of 22 °C, holding pressure of 55 MPa, holding time of 30 s, and injection time of 3 s.
(3) Optimized numerical simulation verification results demonstrate that the maximum warpage deformation of the molded part under the optimal process is 2.224 mm, a 58.03% reduction compared to 5.299 mm under the initial process; the maximum residual stress is 47.42 MPa, a 13.67% reduction compared to 54.93 MPa under the initial process. Both core molding quality indexes are significantly improved and fully meet the engineering application requirements of the plastic part. The relative errors between the numerical simulation results and the algorithm-predicted values are all less than 2%, which verifies the prediction accuracy of the surrogate model and optimization algorithm.
(4) The co-simulation results based on Moldflow-Ansys indicate that the average fiber orientation degree in the main area of the plastic part under the optimal process is about 0.7, and the average tensile modulus along the melt flow direction is 1.85 times that perpendicular to the flow direction. The material exhibits significant mechanical anisotropy, and the spatial distribution of tensile modulus is highly consistent with the fiber orientation distribution. Compared to the traditional isotropic simulation, the co-simulation considering molding history can accurately reflect the regulatory effect of fiber orientation on stress distribution and deformation behavior. The predicted maximum deformation of 4.01 mm and maximum equivalent stress of 43.20 MPa are more consistent with the actual service state of the plastic part. In addition, the deformation and stress of the plastic part under an extreme load of 150 N are far lower than the allowable thresholds, with a sufficient safety margin.
(5) The injection mold trial experimental results demonstrate that the fan face shell produced with the optimized process parameters has good appearance quality without obvious molding defects. The average warpage deformation of eight samples measured by a coordinate measuring machine (CMM) is 2.044 mm, with a relative error of 7.583% compared to the algorithm-predicted value, which further verifies the engineering accuracy and applicability of the process optimization method proposed in this paper.

The RSM-NSGA-II bi-objective process optimization method, the mechanical performance co-simulation process considering molding history, and the full-chain research mode of “simulation optimization—performance analysis—experimental verification” proposed in this paper can provide a complete technical scheme for molding quality control and service performance improvement of large-scale GF-PP injection-molded shells. Nevertheless, this study still has two main limitations that need to be clarified. First, it is a single case study focusing on a flat thin-walled 30% GF-PP fan face shell with specific dimensions and dense stiffener/rib features. For large GF-PP shells with different geometric complexities (such as deep cavities, complex curved surfaces and variable wall thickness structures) and size specifications, the process parameter sensitivity, fiber orientation evolution law and warpage-residual stress coupling mechanism may show significant differences, so the universality of the proposed method needs further verification in more application scenarios. Second, the research results have obvious material specificity. This work only investigates the Extron 3019 HS grade GF-PP composite with a fixed glass fiber mass fraction of 30% and an aspect ratio of 25. For GF-PP materials with different glass fiber contents, aspect ratios, matrix modification types and additive formulations, their rheological properties, fiber orientation kinetics and mechanical anisotropy characteristics will vary greatly. The established response surface surrogate models and co-simulation process need to be recalibrated and adaptively adjusted for different material systems.

For future research directions, the following aspects can be further expanded on the basis of the existing work. (1) Multi-material system optimization: Extend the bi-objective optimization framework proposed in this study to hybrid fiber-reinforced composites (such as glass fiber/basalt fiber, glass fiber/carbon fiber hybrid reinforced polypropylene) and multi-material co-injection systems, and establish a multi-objective optimization method considering interface bonding performance and material matching characteristics to realize the synergistic play of performance advantages of different materials. (2) Fatigue performance and long-term service reliability research: Systematically carry out constant amplitude and variable amplitude cyclic loading fatigue tests on the optimized plastic parts, establish a fatigue life prediction model coupling fiber orientation distribution, residual stress field and environmental factors (temperature, humidity, and ultraviolet radiation), and reveal the influence mechanism of molding history on the fatigue failure behavior and long-term service reliability of GF-PP structural parts. (3) Multi-scale evolution simulation of fiber orientation: Construct a full-chain correlation model of “molding process—microscopic fiber structure—mesoscopic mechanical properties—macroscopic service performance” by combining molecular dynamics simulation, Representative Volume Element (RVE) homogenization method and macro structural mechanics analysis, so as to realize the quantitative prediction and active regulation of material mechanical properties. These extended studies will provide more in-depth and comprehensive theoretical support for the high-performance, lightweight and long-life design of glass fiber-reinforced thermoplastic composite structural parts.

## Figures and Tables

**Table 1:** Material properties and recommended process parameters of Extron 3019 HS.

Glass Fiber Mass Fraction	Fiber Aspect Ratio	Density	Maximum Shear Stress	Maximum Shear Rate	Mold Temperature	Melt Temperature
30%	25	1.14 g/cm^3^	0.25 MPa	100,000 s^−1^	20–60 °C	200–260 °C

**Table 2:** Mesh repair results.

Number of Elements	Maximum Aspect Ratio	Average Aspect Ratio	Minimum Aspect Ratio	Matching Percentage/%	Number of Free Edges
67,440	19.03	2.17	1.16	93.4	0

**Table 3:** Matching relationship between coolant flow velocity and pipe diameter.

Cooling Pipe Diameter *d*/mm	Minimum Velocity *v*/(m·s^−1^)	Volume Flow Rate *V*/(m^3^·min^−1^)
8	1.66	<5.0 × 10^−3^
10	1.32	5.0 × 10^−3^~6.2 × 10^−3^
12	1.10	6.2 × 10^−3^~7.4 × 10^−3^
15	0.87	7.4 × 10^−3^~9.2 × 10^−3^

**Table 4:** Factors and levels of the CCD experiments.

Factor	Level 1 (Low)	Level 2 (Center)	Level 3 (High)
*A*/°C	200	230	260
*B*/°C	20	40	60
*C*/MPa	20	50	80
*D*/s	10	20	30
*E*/s	1	2	3
*F*/s	15	20	25

**Table 5:** Parameters of the TTI-1000SeII injection molding machine.

Item	Parameter
Screw diameter/mm	90
Theoretical injection volume/cm^3^	2799
Injection pressure/MPa	165
Injection rate/(cm^3^·s^−1^)	651
Plasticizing capacity/(g·s^−1^)	93
Ejection stroke/mm	300

**Table 6:** Results of CCD Experimental Design (15 sets of representative data).

No.	*A*	*B*	*C*	*D*	*E*	*F*	Warpage Deformation/mm	Residual Stress/MPa	Run Type
2	230	40	50	20	2	20	3.088	51.51	Center Point
8	230	40	50	20	2	20	3.088	51.51	Center Point
10	230	40	50	20	2	20	3.088	51.51	Center Point
23	230	40	50	20	2	20	3.088	51.51	Center Point
66	230	40	50	20	2	20	3.088	51.51	Center Point
3	183.047	40	50	20	2	20	3.198	53.75	Axial Point
38	276.953	40	50	20	2	20	3.098	49.22	Axial Point
78	230	71.302	50	20	2	20	3.662	54.29	Axial Point
80	230	8.698	50	20	2	20	2.603	50.57	Axial Point
35	230	40	96.952	20	2	20	2.447	101.00	Axial Point
1	260	60	80	30	1	25	2.799	68.90	Factorial Point
4	260	20	20	10	1	25	4.742	43.71	Factorial Point
9	200	60	80	10	1	15	5.129	85.34	Factorial Point
13	200	20	20	10	1	15	3.675	47.90	Factorial Point
16	200	20	20	30	3	25	3.074	47.96	Factorial Point

**Table 7:** ANOVA results of warpage deformation.

Source	Sum of Squares (SS)	Degrees of Freedom (DF)	Mean Square (MS)	F-Value	*p*-Value
Model	72.41	33	2.19	443.92	<0.0001
*A*	0.005	1	0.005	1.01	0.3192
*B*	0.5607	1	0.5607	113.45	<0.0001
*C*	2.81	1	2.81	568.67	<0.0001
*D*	2.71	1	2.71	548.23	<0.0001
*E*	0.03	1	0.03	6.07	0.0171
*AB*	0.8628	1	0.8628	174.56	<0.0001
*AC*	0.0605	1	0.0605	12.23	0.001
*AD*	2.42	1	2.42	489.91	<0.0001
*AE*	0.0203	1	0.0203	4.1	0.048
*BC*	0.03	1	0.03	6.06	0.0171
*BD*	0.9489	1	0.9489	191.98	<0.0001
*BE*	0.1398	1	0.1398	28.28	<0.0001
*CD*	1.68	1	1.68	340.01	<0.0001
*CE*	0.0005	1	0.0005	0.1036	0.7489
*DE*	0.4292	1	0.4292	86.83	<0.0001
*A* ^2^	0.0001	1	0.0001	0.0114	0.9155
*B* ^2^	0.0003	1	0.0003	0.0571	0.812
*C* ^2^	0.5885	1	0.5885	119.07	<0.0001
*D* ^2^	2.03	1	2.03	409.86	<0.0001
*ABC*	0.1503	1	0.1503	30.4	<0.0001
*ABD*	1.31	1	1.31	264.26	<0.0001
*ABE*	0.0331	1	0.0331	6.69	0.0125
*ACD*	0.0083	1	0.0083	1.68	0.2006
*ACE*	0.0067	1	0.0067	1.36	0.2481
*ADE*	0.0207	1	0.0207	4.19	0.0458
*BCD*	0.002	1	0.002	0.3984	0.5307
*BDE*	0.0985	1	0.0985	19.93	<0.0001
*CDE*	0.0101	1	0.0101	2.05	0.1583
*A* ^2^ *B*	0.0307	1	0.0307	6.2	0.016
*A* ^2^ *C*	1.38	1	1.38	279.15	<0.0001
*A* ^2^ *D*	0.0028	1	0.0028	0.5599	0.4577
*A* ^2^ *E*	0.003	1	0.003	0.6073	0.4394
*AB* ^2^	0.0726	1	0.0726	14.68	0.0003
Residual	0.257	52	0.0049		
Lack of Fit	0.257	43	0.006		

**Table 8:** ANOVA results of residual stress.

Source	Sum of Squares (SS)	Degrees of Freedom (DF)	Mean Square (MS)	F-Value	*p*-Value
Model	16,747.56	35	478.5	265.99	<0.0001
*A*	10.26	1	10.26	5.7	0.0207
*B*	6.92	1	6.92	3.85	0.0554
*C*	1458	1	1458	810.46	<0.0001
*D*	0.0313	1	0.0313	0.0174	0.8957
*E*	0.5513	1	0.5513	0.3064	0.5823
*AB*	33.76	1	33.76	18.76	<0.0001
*AC*	259.53	1	259.53	144.27	<0.0001
*AD*	39.82	1	39.82	22.13	<0.0001
*AE*	0.021	1	0.021	0.0117	0.9143
*BC*	819.1	1	819.1	455.32	<0.0001
*BD*	54.32	1	54.32	30.19	<0.0001
*BE*	61.39	1	61.39	34.12	<0.0001
*CD*	105.99	1	105.99	58.92	<0.0001
*CE*	14.4	1	14.4	8.01	0.0067
*DE*	125.44	1	125.44	69.73	<0.0001
*A* ^2^	2.15	1	2.15	1.19	0.2797
*B* ^2^	0.0001	1	0.0001	0.0001	0.9941
*C* ^2^	1103.36	1	1103.36	613.33	<0.0001
*D* ^2^	2.52	1	2.52	1.4	0.2418
*E* ^2^	3.78	1	3.78	2.1	0.1536
*ABC*	46.89	1	46.89	26.06	<0.0001
*ABD*	1.56	1	1.56	0.8651	0.3568
*ABE*	2.67	1	2.67	1.48	0.2293
*ACD*	21.32	1	21.32	11.85	0.0012
*ACE*	0.7098	1	0.7098	0.3946	0.5328
*ADE*	8.57	1	8.57	4.76	0.0338
*BCD*	32.35	1	32.35	17.98	<0.0001
*BCE*	79.52	1	79.52	44.2	<0.0001
*BDE*	39.47	1	39.47	21.94	<0.0001
*CDE*	127.97	1	127.97	71.14	<0.0001
*A* ^2^ *B*	31.45	1	31.45	17.48	0.0001
*A* ^2^ *C*	80.8	1	80.8	44.91	<0.0001
*A* ^2^ *D*	9.13	1	9.13	5.07	0.0287
*A* ^2^ *E*	2.74	1	2.74	1.52	0.223
*AB* ^2^	11.07	1	11.07	6.15	0.0165
Residual	89.95	50	1.8		
Lack of Fit	89.95	41	2.19		

**Table 9:** The 95% prediction intervals for response surface models at the central design point.

Validation Point Type	Response Index	Predicted Mean Value	Observed Value	Relative Error	95% Prediction Interval	Interval Coverage
Center Replicate Point (5 replicates)	Warpage Deformation (mm)	3.103	3.088	0.484%	[2.958, 3.249]	Yes
	Residual Stress (MPa)	51.760	51.51	0.483%	[48.974, 54.545]	Yes

**Table 10:** Comparison between measured maximum warpage deformation and algorithm-predicted results.

Measured Maximum Warpage Deformation of Samples/mm								Average Value/mm	Predicted Value/mm	Relative Error/%
1	2	3	4	5	6	7	8			
2.041	2.111	2.224	2.112	1.852	1.947	1.851	2.215	2.044	2.199	7.583

## Data Availability

The original contributions presented in this study are included in the article and Supplementary Materials. The Moldflow simulation files (.sdy), Ansys structural analysis files (.wbpj), NSGA-II algorithm code (MATLAB .m files), and raw experimental measurement data are available from the corresponding author upon reasonable request.

## Supplementary Materials

The following supporting information can be downloaded at: https://www.mdpi.com/article/10.3390/polym18111373/s1, Table S1: Complete 86-run Central Composite Design (CCD) experimental dataset for injection molding process parameters and cor-responding response values (warpage deformation and residual stress).
