# Supplementary material for: Research on Bi-Objective Optimization of Injection Molding Process and Mechanical Anisotropy of Glass Fiber-Reinforced Polypropylene Fan Face Shell Based on RSM and NSGA-II
Source: Polymers (Basel). 2026 May 31;18(11):1373. doi: 10.3390/polym18111373 (PMC13259024; doi:10.3390/polym18111373)
Supplement: Supplementary file 1 [file polymers-18-01373-s001.zip › polymers-4302286-supplementary.pdf]

polymers

MDPI

*Article*

# Research on Bi-Objective Optimization of Injection Molding Process and Mechanical Anisotropy of Glass Fiber-Reinforced Polypropylene Fan Face Shell Based on RSM and NSGA-II

**Ming Yang [1], Sailong Yan [1], Jubao Liu [1]*, Feng Li [2], Jianfeng Yao [2] and Yasheng Li [1]**

1. Mechanical Science and Engineering College, Northeast Petroleum University, Daqing 163318, China
2. Hongfeng Intelligent Manufacturing (Shenzhen) Co., Ltd., Shenzhen 518117, China

## Supplementary Materials

The following supporting information can be downloaded at: https://www.mdpi.com/article/10.3390/polym18111373/s1.

**Table S1.** Complete 86-run Central Composite Design (CCD) experimental dataset for injection molding process parameters and corresponding response values (warpage deformation and residual stress).

| No. | *A* | *B* | *C* | *D* | *E* | *F* | Warpage Deformation /mm | Residual Stress/MPa |
|---|---|---|---|---|---|---|---|---|
| 1 | 260 | 60 | 80 | 30 | 1 | 25 | 2.799 | 68.90 |
| 2 | 230 | 40 | 50 | 20 | 2 | 20 | 3.088 | 51.51 |
| 3 | 183.047 | 40 | 50 | 20 | 2 | 20 | 3.198 | 53.75 |
| 4 | 260 | 20 | 20 | 10 | 1 | 25 | 4.742 | 43.71 |
| 5 | 200 | 60 | 80 | 30 | 3 | 15 | 2.846 | 84.92 |
| 6 | 260 | 20 | 80 | 10 | 1 | 25 | 4.763 | 74.02 |
| 7 | 230 | 40 | 50 | 35.650 | 2 | 20 | 2.887 | 51.28 |
| 8 | 230 | 40 | 50 | 20 | 2 | 20 | 3.088 | 51.51 |
| 9 | 200 | 60 | 80 | 10 | 1 | 15 | 5.129 | 85.34 |
| 10 | 230 | 40 | 50 | 20 | 2 | 20 | 3.088 | 51.51 |
| 11 | 230 | 40 | 50 | 20 | 2 | 20 | 3.088 | 51.51 |
| 12 | 260 | 60 | 80 | 30 | 1 | 15 | 2.785 | 67.59 |
| 13 | 200 | 20 | 20 | 10 | 1 | 15 | 3.675 | 47.90 |
| 14 | 230 | 40 | 50 | 20 | 0.435 | 20 | 3.266 | 51.70 |
| 15 | 260 | 60 | 80 | 10 | 3 | 15 | 4.913 | 86.59 |
| 16 | 200 | 20 | 20 | 30 | 3 | 25 | 3.074 | 47.96 |
| 17 | 200 | 60 | 20 | 30 | 1 | 25 | 3.650 | 48.49 |
| 18 | 200 | 20 | 80 | 10 | 1 | 25 | 3.486 | 62.75 |
| 19 | 260 | 20 | 80 | 10 | 3 | 25 | 4.254 | 67.87 |
| 20 | 260 | 60 | 20 | 10 | 1 | 15 | 5.129 | 45.39 |
| 21 | 200 | 20 | 20 | 30 | 3 | 15 | 3.074 | 47.96 |

| | | | | | | | | |
|---|---|---|---|---|---|---|---|---|
| 22 | 260 | 20 | 20 | 10 | 1 | 15 | 4.742 | 43.71 |
| 23 | 230 | 40 | 50 | 20 | 2 | 20 | 3.088 | 51.51 |
| 24 | 200 | 60 | 20 | 30 | 1 | 15 | 3.65 | 48.49 |
| 25 | 230 | 40 | 50 | 20 | 2 | 20 | 3.088 | 51.51 |
| 26 | 230 | 40 | 50 | 20 | 2 | 20 | 3.088 | 51.51 |
| 27 | 260 | 20 | 80 | 10 | 3 | 15 | 4.253 | 67.87 |
| 28 | 200 | 20 | 80 | 30 | 1 | 15 | 2.377 | 61.13 |
| 29 | 200 | 60 | 80 | 30 | 1 | 15 | 2.866 | 67.34 |
| 30 | 200 | 60 | 80 | 10 | 3 | 25 | 4.755 | 78.69 |
| 31 | 260 | 20 | 80 | 30 | 1 | 15 | 2.215 | 66.31 |
| 32 | 260 | 60 | 20 | 30 | 3 | 25 | 3.456 | 43.68 |
| 33 | 260 | 20 | 80 | 30 | 3 | 15 | 2.178 | 65.94 |
| 34 | 260 | 60 | 20 | 30 | 1 | 15 | 3.543 | 43.84 |
| 35 | 230 | 40 | 96.952 | 20 | 2 | 20 | 2.447 | 101.00 |
| 36 | 200 | 60 | 80 | 30 | 1 | 25 | 2.865 | 67.34 |
| 37 | 200 | 20 | 20 | 30 | 1 | 15 | 3.196 | 47.96 |
| 38 | 276.953 | 40 | 50 | 20 | 2 | 20 | 3.098 | 49.22 |
| 39 | 260 | 60 | 20 | 30 | 1 | 25 | 3.543 | 43.84 |
| 40 | 200 | 60 | 20 | 10 | 1 | 15 | 5.183 | 48.50 |
| 41 | 260 | 20 | 20 | 30 | 1 | 15 | 2.736 | 43.68 |
| 42 | 200 | 60 | 20 | 10 | 3 | 15 | 4.725 | 47.82 |
| 43 | 200 | 20 | 20 | 10 | 1 | 25 | 3.675 | 47.90 |
| 44 | 230 | 40 | 50 | 20 | 3.565 | 20 | 3.021 | 50.65 |
| 45 | 260 | 60 | 80 | 30 | 3 | 15 | 2.706 | 80.30 |
| 46 | 200 | 60 | 80 | 10 | 1 | 25 | 5.136 | 85.34 |
| 47 | 200 | 20 | 80 | 10 | 1 | 15 | 3.485 | 62.75 |
| 48 | 260 | 60 | 80 | 10 | 1 | 25 | 4.892 | 84.28 |
| 49 | 230 | 40 | 50 | 20 | 2 | 27.820 | 3.075 | 51.28 |
| 50 | 260 | 60 | 20 | 10 | 3 | 15 | 5.120 | 45.51 |
| 51 | 260 | 20 | 20 | 10 | 3 | 15 | 4.011 | 44.05 |
| 52 | 200 | 20 | 80 | 10 | 3 | 25 | 2.962 | 59.27 |
| 53 | 200 | 20 | 20 | 10 | 3 | 25 | 3.263 | 47.96 |
| 54 | 200 | 20 | 80 | 10 | 3 | 15 | 2.961 | 59.27 |
| 55 | 260 | 20 | 80 | 30 | 1 | 25 | 2.215 | 66.31 |

| | | | | | | | | |
|---|---|---|---|---|---|---|---|---|
| 56 | 260 | 60 | 20 | 10 | 1 | 25 | 5.129 | 45.39 |
| 57 | 200 | 20 | 80 | 30 | 3 | 15 | 2.267 | 62.29 |
| 58 | 200 | 60 | 20 | 10 | 1 | 25 | 5.183 | 48.50 |
| 59 | 200 | 60 | 80 | 30 | 3 | 25 | 2.845 | 84.92 |
| 60 | 260 | 20 | 20 | 30 | 3 | 15 | 2.766 | 44.05 |
| 61 | 200 | 60 | 80 | 10 | 3 | 15 | 4.752 | 78.69 |
| 62 | 260 | 60 | 20 | 10 | 3 | 25 | 5.120 | 45.51 |
| 63 | 260 | 20 | 20 | 30 | 3 | 25 | 2.767 | 44.07 |
| 64 | 200 | 20 | 80 | 30 | 3 | 25 | 2.266 | 60.73 |
| 65 | 260 | 20 | 20 | 10 | 3 | 25 | 4.011 | 44.05 |
| 66 | 230 | 40 | 50 | 20 | 2 | 20 | 3.088 | 51.51 |
| 67 | 230 | 40 | 50 | 20 | 2 | 12.175 | 3.075 | 51.28 |
| 68 | 230 | 40 | 50 | 20 | 2 | 20 | 3.088 | 51.51 |
| 69 | 230 | 40 | 50 | 20 | 2 | 20 | 3.088 | 51.51 |
| 70 | 260 | 60 | 20 | 30 | 3 | 15 | 3.461 | 43.75 |
| 71 | 260 | 60 | 80 | 10 | 1 | 15 | 4.845 | 86.73 |
| 72 | 230 | 40 | 3.047 | 20 | 2 | 20 | 4.818 | 47.00 |
| 73 | 200 | 60 | 20 | 30 | 3 | 25 | 3.725 | 47.82 |
| 74 | 230 | 40 | 50 | 4.349 | 2 | 20 | 5.215 | 51.53 |
| 75 | 260 | 60 | 80 | 10 | 3 | 25 | 4.918 | 86.59 |
| 76 | 200 | 20 | 20 | 30 | 1 | 25 | 3.191 | 47.90 |
| 77 | 200 | 20 | 80 | 30 | 1 | 25 | 2.377 | 60.76 |
| 78 | 230 | 71.302 | 50 | 20 | 2 | 20 | 3.662 | 54.29 |
| 79 | 260 | 60 | 80 | 30 | 3 | 25 | 2.704 | 80.30 |
| 80 | 230 | 8.698 | 50 | 20 | 2 | 20 | 2.603 | 50.57 |
| 81 | 200 | 20 | 20 | 10 | 3 | 15 | 3.263 | 47.96 |
| 82 | 200 | 60 | 20 | 10 | 3 | 25 | 4.725 | 47.82 |
| 83 | 260 | 20 | 20 | 30 | 1 | 25 | 2.736 | 43.68 |
| 84 | 260 | 20 | 80 | 30 | 3 | 25 | 2.178 | 65.94 |
| 85 | 260 | 20 | 80 | 10 | 1 | 15 | 4.759 | 74.02 |
| 86 | 200 | 60 | 20 | 30 | 3 | 15 | 3.725 | 47.82 |
